# Diversity and Selection of Surface Marine Microbiomes in the Atlantic-Influenced Arctic

**DOI:** 10.3389/fmicb.2022.892634

**Published:** 2022-07-14

**Authors:** Nerea J. Aalto, Hannah D. Schweitzer, Stina Krsmanovic, Karley Campbell, Hans C. Bernstein

**Affiliations:** ^1^Faculty of Biosciences, Fisheries, and Economics, UiT—The Arctic University of Norway, Tromsø, Norway; ^2^The Arctic Centre for Sustainable Energy, UiT—The Arctic University of Norway, Tromsø, Norway

**Keywords:** diatoms, Arctic Ocean, sea-ice, null model, temperature, Atlantification, Nansen Basin

## Abstract

Arctic marine environments are experiencing rapid changes due to the polar amplification of global warming. These changes impact the habitat of the cold-adapted microbial communities, which underpin biogeochemical cycles and marine food webs. We comparatively investigated the differences in prokaryotic and microeukaryotic taxa between summer surface water microbiomes sampled along a latitudinal transect from the ice-free southern Barents Sea and into the sea-ice-covered Nansen Basin to disentangle the dominating community (ecological) selection processes driving phylogenetic diversity. The community structure and richness of each site-specific microbiome were assessed in relation to the physical and biogeochemical conditions of the environment. A strong homogeneous deterministic selection process was inferred across the entire sampling transect *via* a phylogenetic null modeling approach. The microbial species richness and diversity were not negatively influenced by northward decreasing temperature and salinity. The results also suggest that regional phytoplankton blooms are a major prevalent factor in governing the bacterial community structure. This study supports the consideration that strong homogeneous selection is imposed across these cold-water marine environments uniformly, regardless of geographic assignments within either the Nansen Basin or the Barents Sea.

## Introduction

Surface waters of the Arctic marine system are inhabited by cold-adapted microbial taxa that persist under high availably of organic carbon sources and seasonal extremes in light (Bussmann and Kattner, 2000; Riedel et al., 2006). These unique microbiomes, encompassing both prokaryotic and microeukaryotic taxa, are in transition as Arctic habitats change from global warming and Atlantification. Environmental alterations to the Arctic Ocean include decreases in sea-ice extent and volume (Kwok, 2018) and increases in surface water temperature in regions influenced by Atlantic inflow (Polyakov et al., 2017). To understand the ecological implications of these changes, it is important to investigate the diversity and structure of microbial communities because these cold-adapted microbiomes have an important role in underpinning biogeochemical cycles (Falkowski et al., 2008) and marine food webs. Pelagic prokaryotes (Bacteria and Archaea) and microeukaryotes (microalgae and heterotrophic protists) comprise a wide range of functional types within cold-water marine ecosystems and have distinct environments or nutrient requirements (Quere et al., 2005; Cao et al., 2020). A growing body of evidence shows that environmental disturbances alter the diversity and structure of these microbial communities (Barton et al., 2016; Assmy et al., 2017; Orkney et al., 2020; Oziel et al., 2020), which can impact ecosystem functioning and marine carbon sequestration *via* deep-sea carbon export (Assmy et al., 2017; Schweitzer et al., 2021).

Due to the importance of marine microbiomes and the rapid development of molecular tools in the past decade, there have been increasing numbers of studies investigating environmental and ecophysiological factors selecting for a regional to global scale microbial diversity and richness. For example, several studies have revealed a decrease in both prokaryote and microeukaryote taxonomic diversity and richness with increasing latitude at the global scale (Fuhrman et al., 2008; Ibarbalz et al., 2019; Salazar et al., 2019; Martin et al., 2021). This observed pattern has been linked to the latitudinal relationship with temperature, suggesting that temperature is one of the most important environmental factors driving the species richness and taxonomic composition of upper ocean microbial communities. Multiple studies have also illustrated biogeographical differentiation in the taxonomic and transcriptomic diversity of microbial communities between comparatively cold (polar) and warm (non-polar) waters, as well as unique algal microbiome co-occurrence networks and rates of net primary production (Behrenfeld et al., 2006; Ibarbalz et al., 2019; Salazar et al., 2019; Martin et al., 2021). Many of these differences are suggested to occur in the northern hemisphere around an annual-mean isotherm of 14–15°C as an ecological boundary or “breakpoint,” potentially selecting for thermal tolerance limits and affecting the kinetics of metabolism (Thomas et al., 2012).

The availability of nutrients and photosynthetically active radiation (PAR), which are both linked to the mixed layer depth (MLD), have been implicated to control regional differences in the phytoplankton community structure, more so than temperature (Barton et al., 2016; Oziel et al., 2017; Aalto et al., 2021). Phytoplankton blooms are also known to exert major influences on localized marine microbiomes (Cirri and Pohnert, 2019). Microalgae form complex interactions with prokaryotes that utilize and remineralize dissolved organic matter released by phytoplankton and in some cases exchange metabolites back to their photoautotrophic hosts (Buchan et al., 2014). Temperate and cold-water bacterial community structure have been shown to vary with the species composition of phytoplankton blooms, as well as within the dynamic time course of a bloom (Teeling et al., 2012; Fadeev et al., 2018; Rapp et al., 2018). Thus, the surface water bacterioplankton community structure is hypothesized to largely be determined by environmental factors associated with phytoplankton bloom phenology (Fadeev et al., 2018).

The Barents Sea, a relatively shallow and large shelf sea, is well described with respect to hydrography, seasonal phytoplankton community, and primary production (e.g., Loeng, 1991; Wassmann et al., 2006a; Degerlund and Eilertsen, 2010). However, the spatial heterogeneity of prokaryote taxonomic diversity and community structure is poorly documented. Typically, the annual primary production is highest in the ice-free and marginal ice zones of the Barents Sea (Wassmann et al., 2006a). Diatoms are highly abundant in these locations and contribute to high dissolved organic carbon (DOC) production and vertical carbon flux (Wassmann et al., 2006b; Matrai et al., 2007). The influence of this seasonally high diatom abundance and associated DOC on co-occurring microbial communities remains poorly understood within the Barents Sea (Wassmann et al., 2006a). In studies from other areas, a small number of genera from bacteria phyla Proteobacteria and Bacteroidetes have been observed to respond to a diatom bloom, but it has been also found that the associated microbiome can vary between and within diatom species (Amin et al., 2012; Teeling et al., 2012; Ajani et al., 2018). Overall, the spring phytoplankton blooms in the Barents Sea follow a transition from diatoms such as centric *Chaetoceros* spp. and *Thalassiosira* spp. and haptophyte algae *Phaeocystis* sp. domination to a more diverse group of flagellates and dinoflagellates (Wassmann et al., 2006a; Degerlund and Eilertsen, 2010).

In contrast to the Barents Sea, the Nansen Basin in the high Arctic Ocean is characterized by deep topography and surface water temperature near the freezing point (~-1.5°C). Phytoplankton production in the ice-covered Arctic is comparatively low to the open ocean due to restricted light availability. Despite the low production in the Nansen Basin region, the surface water comprises a diverse group of autotrophic and heterotrophic flagellates and dinoflagellates (Rapp et al., 2018; de Sousa et al., 2019). However, diatom and *Phaeocystis* sp. dominated blooms have been reported beneath sea ice during times of enhanced light transmission to the upper ocean due to the formation of leads and melt ponds (Arrigo et al., 2012; Assmy et al., 2017).

Heat transfer to the Barents Sea and the Eurasian Basin of the Arctic Ocean occurs by the northward inflow of North Atlantic water. The Barents Sea and the Nansen Basin are influenced by Atlantic water as the Norwegian Atlantic current enters the Barents Sea and the Fram Strait (Rudels et al., 1994). However, surface waters in the Nansen Basin are characterized by less saline and colder Polar Surface water in comparison to Atlantic water (Anderson et al., 1989). The influence of Atlantic water has increased substantially during the last decades, by a phenomenon known as Atlantification (Oziel et al., 2017).

Exclusive focus between microbial taxa and regional variation of environmental factors, as is most often the case in previous studies, can impede the comprehensive understanding of underlying fundamental community (ecological) selection processes governing the phylogenetic diversity (Stegen et al., 2012, 2013). Only a small number of studies, none of them in the Arctic (as we are aware), have applied this concept and related statistical tools such as null modeling-based randomization to infer the spatial and/or temporal influence of stochastic and deterministic selection processes on surface water microbiomes (e.g., Allen et al., 2020). Stochasticity refers to weak (neutral) selection so that the community composition is not determined according to the environmental fitness of the members but rather dispersion rate and randomness (Vellend, 2010; Dini-Andreote et al., 2015; Evans et al., 2017). Whereas abiotic and biotic factors in deterministic selection processes act as a strong selection force for species with different fitness (Vellend, 2010).

As the Barents Sea, and to a lesser extent the Nansen Basin, are influenced by Atlantic water, it is important to investigate how ongoing Atlantification and associated warming of surface waters affects the marine microbiomes. In this study, we surveyed surface water (0.5 and/or 20 m) microbial community structure during the summer using rRNA gene amplicon sequencing across a latitudinal transect spanning from the southern Barents Sea to the sea-ice-covered Nansen Basin. Four main research questions were addressed: (1) How does the surface water microbial community structure change along the transect and what are the differentially represented taxa designating these areas? (2) What are the main environmental factors linked to the differences in microbial community composition and how does the relative abundance of diatoms impact the prokaryotic structure? (3) How does the change in salinity from sea-ice melt in summer influence the microbial community beneath the sea-ice bottom in the Nansen Basin? and (4) Do stochastic or deterministic processes play greater roles in the ecological selection that influence the prokaryote and microeukaryote members of these microbiomes?

## Methods

### Study Area

**Figure 1:**
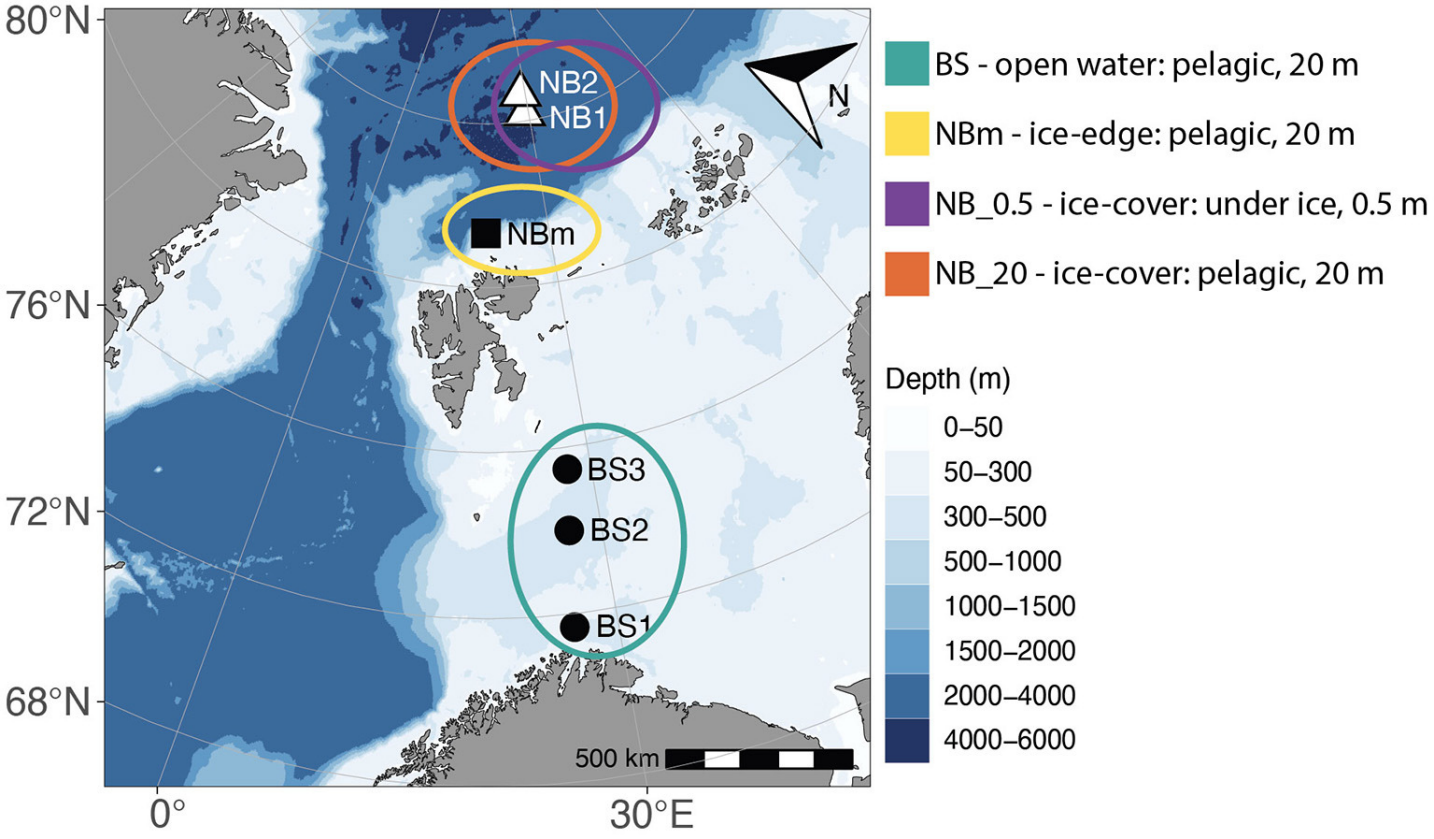
A Map showing the geographical location of sample stations—south and north of Svalbard. Samples were collected from surface water, 20 m, at all stations (•, Barents Sea stations: BS1, BS2, and BS3; ■, sea-ice edge station in Nansen Basin margin: NBm; ▵, sea-ice covered stations in Nansen Basin: NB1 and NB2). In addition, at NB1 and NB2 stations, samples were collected directly under sea ice,0.5 m. The colored circles represent sample locations that were grouped based on the environment type: BS (green), Barents Sea communities in the open water environment; NBm (yellow), Nansen Basin margin community in sea-ice edge environment; NB_0.5 (purple), Nansen Basin communities in interface environment between sea-ice and surface water; and NB_20 (orange), Nansen Basin communities in sea-ice covered environment.

This study was conducted across a transect from 71.7 to 84.7° north on the *Bioprospecting 2019* expedition with RV Kronprins Haakon (July 04–18 2019). Sampling took place from the ice-free southern Barents Sea (Nord Cape Bank) into the ice-covered Arctic Ocean (Nansen Basin), with a total of six sampling stations: BS1, BS2, and BS3 along the open-water Barents Sea; NBm near the ice-edge over the slope between deep Nansen Basin and shallower Barents Sea in the region referred to as the Nansen Basin margin, and NB1 and NB2 in the sea-ice covered Nansen Basin (Figure 1; Supplementary Table 1; Supplementary Figure 1).

### Water Sampling and Data Collection

At the Barents Sea stations (BS1, BS2, and BS3), measurements of temperature, salinity, and *in vivo* fluorescence through the water column were taken with a SeaBird 9–11 plus (Seabird Scientific, US) conductivity-temperature-depth (CTD) sensor. In the Nansen Basin (NB1 and NB2) and the Nansen Basin margin (NBm) stations, CTD casts were taken down to 50–60 m with a hand-held AML oceanographic X2 electronic CTD sensor (AML Oceanographic, Canada). Water samples were collected from fixed sampling depths of 20 m at all stations and at 0.5 m (directly under sea-ice) at NB1 and NB2 stations (hereafter these samples are referred to as NB1_0.5, NB1_20, NB2_0.5, and NB2_20). The samples from 20 m were collected using a rosette sampler, and the sample water was drawn from several (between 3 and 5) parallelly mounted Niskin samplers (10 L) at open water Barents Sea stations (BS1, BS2, and BS3), while a Niskin sampler (2 L) was manually deployed multiple times (between 10 and 20 casts) through the sea ice at Nansen Basin stations (NB1 and NB2) and over the vessel deck in the vicinity of the ice-edge at NBm station (Nansen Basin margin). Subsamples were collected for analysis of total dissolved inorganic carbon (DIC), dissolved organic carbon (DOC), *in vitro* chlorophyll *a* (chl *a*), and inorganic macronutrients of nitrate (${\text{NO}}_{3}^{-}$), phosphate (${\text{PO}}_{4}^{3-}$), and silicic acid (Si(OH)_4_). Seawater for microbial biomass both for prokaryotic and eukaryotic community investigation was first passed through a 150 μm zooplankton mesh into three clean (aseptically prepared) 20 L carboys and thereafter biological replicates of six were filtered onto 0.22 μm polycarbonate filters (Whatman) *via* vacuum filtration. The filtered volume per filter was 600 ml. Filters were immediately flash-frozen in liquid N_2_ and stored onboard at −80°C. All filtering equipment and ship laboratory spaces were disinfected with 2% bleach prior to and between samples to prevent cross-contamination.

### Sample Analyses

DIC samples were drawn into borosilicate glass bottles *via* silicon tubing and preserved immediately after sampling with saturated mercuric chloride (0.02% of the total volume corresponding to the final concentration of 0.0015%). Samples were stored in dark at +6°C until they were sent to the accredited laboratory of the Norwegian Institute for Water Research (NIVA) 10 months after. DIC was analyzed with VINDTA 3C (Marianda, Germany), and coulometric titration was deployed using a UIC CM5015 coulometer. Samples were warmed up to ~25°C prior to measurement. Certified reference material for seawater DIC from batch 169 (A. Dickson, Scripps Institution of Oceanography) was measured two times daily. The procedure follows the recommended SOP: “*SOP 2: Determination of total dissolved inorganic carbon in seawater*” (Dickson et al., 2007).

Samples for Si(OH)_4_ and ${\text{PO}}_{4}^{3-}$ were filtered immediately after sampling through pre-combusted (450°C) GF/C glass microfiber filters (Whatman) into 50 ml polypropylene Falcon tubes. These nutrients were analyzed in subsample triplicates spectrophotometrically onboard, using Spectroquant (Merck Millipore, US) kits, specifically for each nutrient, whereas ${\text{NO}}_{3}^{-}$ samples were frozen and stored at −20 °C and analyzed 2 months later at the University of Tromsø, Norway. As low ${\text{NO}}_{3}^{-}$ concentration in seawater is difficult to measure due to its reduction potential, it was analyzed by applying a cadmium reduction column where ${\text{NO}}_{3}^{-}$ is reduced to nitrite (${\text{NO}}_{2}^{-}$) (Strickland and Parsons, 1972), and thereafter, the ${\text{NO}}_{2}^{-}$ kit was used and ${\text{NO}}_{3}^{-}$ concentration (${\text{NO}}_{2}^{-}$ is not included to ${\text{NO}}_{3}^{-}$ values) was recalculated from the predetermined standard curve. Quartz cuvettes (5 mL) were used to determine the nutrient concentrations using a spectrometer (UV/Visible spectrometer, UV-6300PC, VWR, US). The absorbance of ${\text{NO}}_{2}^{-}$ was measured at 541 nm, Si(OH)_4_ at 822 nm, and ${\text{PO}}_{4}^{3-}$ at 840 nm.

Disposable polyethersulfone 0.2 μm membrane filter (VWR Sterile Syringe Filter) together with disposable syringes (60 ml) were used to filter DOC samples into acid-washed and pre-combusted (450°C) glass vials of 60 ml (Assistant, Germany) and thereafter stored at −20°C. DOC samples were analyzed 15 months later at the University of Tromsø, Norway with a TOC-VCPH analyzer (Shimadzu, Japan).

A Turner TD-700 fluorometer (Turner Designs, USA) was used to measure *in vitro* chl *a* and pheophytin concentration 24 h after extraction in 96% ethanol at 4°C (Holm-Hansen and Riemann, 1978). For the chl *a*, the extraction sample volume of 5-200 ml was filtrated in subsample triplicate onto the Whatman GF/C filter.

### Water Type Analysis and Mixed Layer Depth

Different water masses were determined from sampling station temperature and salinity. Specifically, the identification of water masses was based on practical salinity, potential temperature, and potential density values outlined for the Barents Sea (Loeng, 1991) and the north region of Svalbard in the Nansen Basin (Pérez-Hernández et al., 2017). The mixed layer depth (MLD) was determined from each CTD-profile data using potential density. The reference depth of 20 m and density threshold of 0.1 kg m^−3^ were used to define MLD, i.e., depth below 20 m where density first exceeds the criteria threshold. The chosen density criteria are typical for the Arctic region (Peralta-Ferriz and Woodgate, 2015) although smaller criteria of 0.003 kg m^−3^ are also used in spring to avoid possible overestimation (Meyer et al., 2017); however, the criteria were not applied here since the shallowest measured CTD-profile depth at Barents Sea stations was 8–15 m and the method is not very criteria sensitive (Peralta-Ferriz and Woodgate, 2015).

### Amplicon Sequencing

Genomic DNA was extracted from 0.22 μm polycarbonate filters using the DNeasy PowerWater Kit (Qiagen, Carlsbad, CA) and all downstream amplicon sequencing analyses were performed in accordance with the Earth Microbiome Project protocols (Gilbert et al., 2010) with minor modifications in quality checks. Quality checks were run on the PCR products using the DNA 12,000 assay with the Agilent Bioanalyzer (Agilent Technologies, Germany). PCR template-free negative controls were also amplified and sequenced in order to identify background sequences. Sequencing was performed on an Illumina MiSeq instrument (Illumina, San Diego, CA) at Argonne National Laboratory (Lemont, IL, USA). From each station and depth template from biological replicates of six were processed *via* 16S and 18S rRNA gene amplification reactions. The Illumina sequencing primer binding site was used with V4 forward primer (515F) and V4 reverse primer (806R) targeting the hypervariable region of 16S rRNA (Caporaso et al., 2010) and correspondingly V9 forward primer (1391F) and V9 reverse primer (EukBr) were used for 18S rRNA as outlined by the Earth Microbiome Project and modified from Amaral-Zettler et al. (2009).

### Amplicon Sequence Analysis

Trimmed Illumina reads were imported and demultiplexed using a corresponding Barcode file into QIIME2 using the Earth Microbiome Project paired-end flag. All reads were denoised and merged using the DADA2 v2021.2.0 algorithm from within QIIME2 (Callahan et al., 2016). The denoising steps of the DADA2 package pooled, filtered, de-replicated, and chimera-checked all reads. After discarding chimeras and singletons, reads were merged, and amplicon sequence variants (ASVs) were inferred using DADA2. Taxonomy was assigned to each ASV using a 16S and 18S rRNA gene self-trained classifier from the SILVA database v138.1 (Quast et al., 2012; Yilmaz et al., 2014). Classifiers were trained using RESCRIPt (Robeson et al., 2020). The phylogenetic rooted tree was generated using the MAFFT algorithm from within QIIME2 and was used for downstream diversity analysis.

### Diversity Analysis

Downstream analysis was completed in R (R Core Team, 2021), using primarily the “microeco” (Liu et al., 2021) and “vegan” packages (Oksanen et al., 2020). From 16S data, reads not assigned to their expected kingdom (Archaea and Bacteria) were removed along with all mitochondria and chloroplast assignments. ASVs belonging to the kingdom of Archaea and Bacteria and phyla of *Vertebrata, Arthropoda, Cnidaria Echinodermata*, and *Mollusca* were removed from 18S data. After removing unwanted taxa, the number of ASVs decreased from 2,179 to 1,731 in the 16S dataset and from 3,052 to 1,812 in the 18S dataset. Samples were rarified to an even depth of 29,746 and 74,750 reads per 16S and 18S sample, respectively, based on minimum sample depths as all the analyzed samples reached asymptote (Supplementary Figure 2). This reduced the total number of ASVs from 1731 to 1505 in the 16S data set and from 1812 to 1752 in the 18S data set. Alpha diversity was quantified by using counts of observed unique ASVs (species richness) and calculating Faith's phylogenetic diversity (PD) to assess the biodiversity of features inherent in genetic sequences (Faith, 1992; Faith and Baker, 2006). A Tukey test was performed to determine the differences in species richness and Faith's PD between samples. The sampled microbial communities were further grouped according to environment conditions, i.e., sea-ice coverage and sample depth. These groups were as follows: BS as a pelagic ice-free environment including all samples collected from the Barents Sea (3 stations, *n* = 6 per station); NBm as a pelagic ice-edge environment containing a sample from the Nansen Basin margin (1 station, *n* = 6); NB_0.5 as the interface environment between the sea-ice bottom and surface water including samples from 0.5 m in the Nansen Basin (2 stations, *n* = 6 per station); and NB_20 as the pelagic sea-ice environment including samples from 20 m in the Nansen Basin (2 stations, *n* = 6 per station; Figure 1). A Venn diagram was generated to provide any additional information on the distribution of shared and exclusive ASVs between grouped communities (BS, NBm, NB_0.5, and NB_20). Beta diversity was measured by calculating unweighted UniFrac distances which consider the presence/absence of ASVs (Lozupone et al., 2011) across all samples (stations and depths) and visualized through distance-based redundancy analysis (dbRDA) with contextual environmental variables (salinity, temperature, DOC, DIC, Si(OH)_4_, chl *a* and relative abundance of diatoms) followed by permutational multivariate analysis of variance (PERMANOVA) (Anderson, 2001).

### Differential Abundance

Linear discriminant analysis effect size (LEfSe) was used to identify specific taxa (i.e., indicator taxa) which were differentially represented between environmentally grouped communities (Segata et al., 2011). The LEfSe analysis consists of three statistical parts (Segata et al., 2011). First, the significant (*p* < 0.01) differential abundance of taxa between grouped communities was obtained by the non-parametric factorial Kruskal-Wallis sum-rank test. Secondly, significantly different taxa were investigated using the pairwise Wilcoxon rank-sum test to encode biological consistency within the grouped communities. The last step was to assign linear discriminant analysis (LDA) for effect size estimation to determine the order of magnitude difference in abundances and induce the ranking of taxa. The specific LDA score of each taxon is obtained by ranking and scaling the taxa based on relative differences among groups along with the 1–10^6^ interval and by computing the logarithm (base 10) of those taxa (Segata et al., 2011). The threshold of LDA score was set to 4.0 to obtain taxa with higher relevance as many taxa were differentially abundant with a commonly used LDA score of 2. As the indicator taxa are obtained *via* sets of pairwise comparisons, there is no need for multiple testing corrections when more than two groups are used (Segata et al., 2011). The indicator taxa determined by LEfSe analysis were used further to investigate relationships between taxa and environmental factors *via* Pearson's correlation.

### Null Models

Null model analysis was implemented by calculating the beta Mean Nearest Taxon Distance (βMNTD), and the beta Nearest Taxon Index (βNTI) was obtained to infer contributions of stochastic versus deterministic selection forces between grouped communities using the R package “picante” (Kembel et al., 2010). The method is based on phylogenetic distances (mean minimum) to assess if the spatial or temporal phylogenetic turnover in the community composition is significantly higher or lower than that expected by chance (Stegen et al., 2012). First, βMNTD (for observed data) quantifies the phylogenetic distance between communities, which is compared to the mean of the null distribution of βMNTD. The null distribution of βMNTD was generated by randomization (here 1000 times) of the phylogenetic position of ASVs in grouped communities (Stegen et al., 2012). βNTI, that is the difference between observed βMNTD and the mean of the null distribution of βMNTD, was quantified by normalizing the null distribution of βMNTD by its standard deviation (Stegen et al., 2012). Thus, βNTI values are given on the z-score scale and the common interpretation is that values greater than +2 and below −2 indicate the dominance of the deterministic selection processes where variable selection and homogeneous selection are prevailing, respectively. Variable selection infers that phylogenetic turnover between communities is higher than expected by chance and homogeneous selection infers the opposite. The dominance of stochastic processes (i.e., no significant selection occurs when the βNTI values are between −2 and +2) is near-zero (Stegen et al., 2012; Dini-Andreote et al., 2015).

### Data Repository and Reproducible Analyses

All data along with R scripts used for analysis and graphing are available on the Open Science Framework as part of this project: https://osf.io/g8wxc/.

## Results

### Comparing the Physical and Biogeochemical Marine Environments

**Figure 2:**
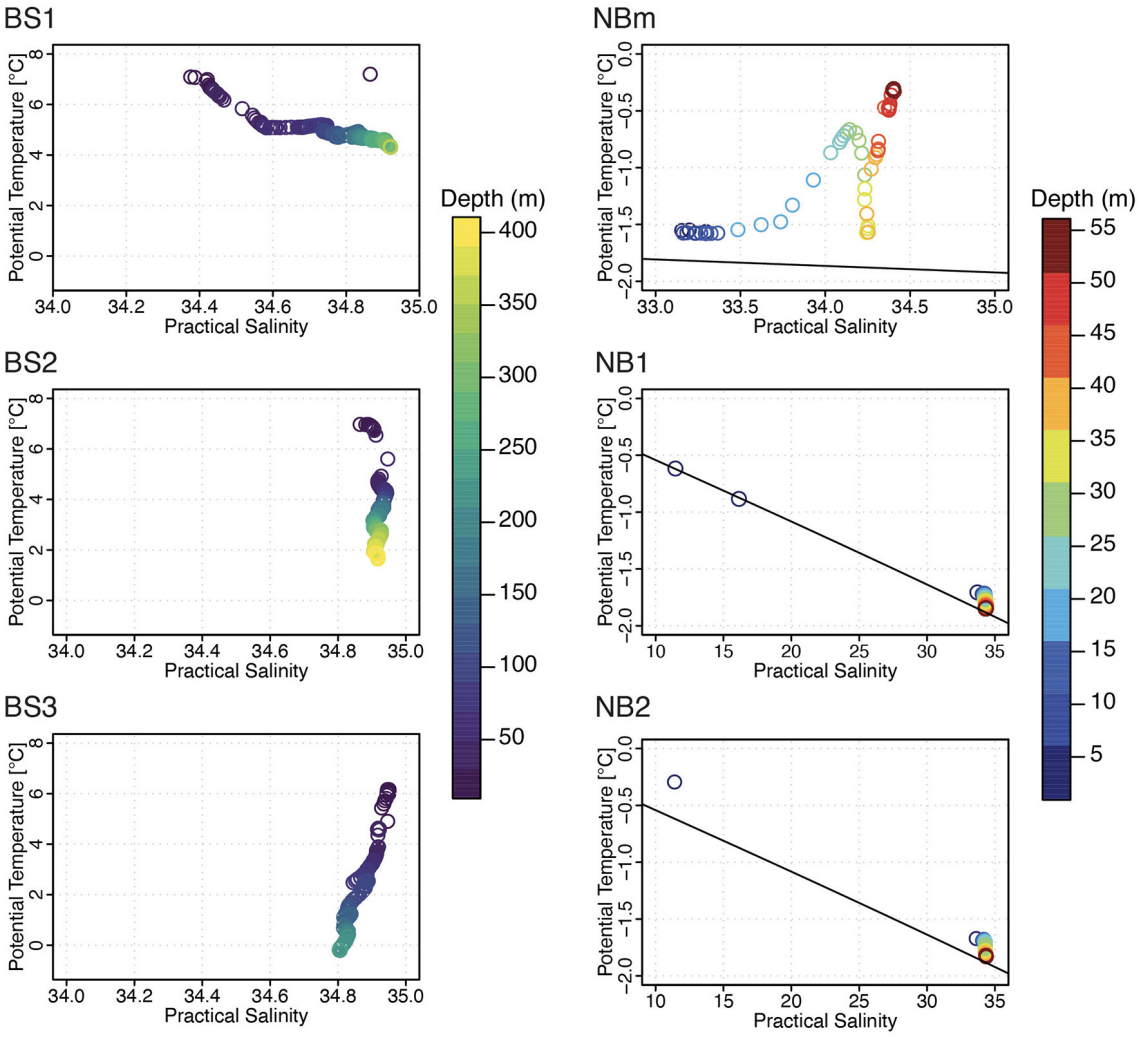
A comparison of the marine physical environment using potential temperature-practical salinity diagram per sampling station. The water column depth (m) is denoted by color legend. Only surface water profile was recorded in NBm, NB1, and NB2 stations. Note the different range of scales at NBm (sea-ice margin) and NB1 and NB2 (sea-ice covered) stations.

Each environment sampled in this study (Figure 1) was unique with respect to one or more factors such as temperature, salinity, nutrients, and/or phytoplankton content (Table 1). According to water type definitions for the Barents Sea and the Arctic Ocean (Loeng, 1991; Pérez-Hernández et al., 2017), the surface layer in the BS1 station was identified to consist of Norwegian coastal current (T > 2°C; S < 34.7), and Atlantic water (T > 3°C; S > 34.9) was the main water type in BS2 station (Figure 2). Whereas, in the BS3 station, the water column properties corresponded to locally formed Polar Front water (T −0.5 − +2°C; S 34.8–35) (Loeng, 1991). The surface water at the representing ice-edge station, NBm, consisted of sea-ice melt and Polar Surface water (T < 0°C). This was similar to the water type for NB1 and NB2 stations in the Nansen Basin where, in addition, a sharp and clearly distinct freshwater lens (S < 16.2) was found at the surface above 2 m induced by sea-ice melt (Figure 2) (Pérez-Hernández et al., 2017). The MLD became shallower from the southern Barents Sea to the Nansen Basin margin, and in the Nansen Basin, the surface water below the low-salinity layer was homogeneous with respect to temperature and salinity (Supplementary Figures 3, 4).

**Table 1:** Summary of the measured and analyzed physical and biogeochemical environmental factors (mean ± standard deviation) at the depth of sampled microbial communities.

**Station**	**BS1**	**BS2**	**BS3**	**NBm**	**NB1**		**NB2**	
**Sample ID/measurement**	**BS1**	**BS2**	**BS3**	**NBm**	**NB1_0.5**	**NB1_20**	**NB2_0.5**	**NB2_20**
	**20 m**	**20 m**	**20 m**	**20 m**	**0.5 m**	**20 m**	**0.5 m**	**20 m**
Sea-ice cover (%)	0	0	0	~50	~85	~85	~90	~90
Temperature^a^	6.9	6.9	6.1	−1.1	−0.5	−1.8	−0.03	−1.2
Salinity^b^	34.4	35.0	35.0	33.9	10.9	34.3	8.6	34.3
DIC (μmol kg^−1^)	2,073.5	2,097.1	2,089.7	2,075.8	688.8	2,113.2	233.3	2124.7
DOC (mg L^−1^)	28.3	31.8	41.9	34.0	24.6	28.3	27.6	34.1
${\text{NO}}_{3}^{-}$ (μmol L^−1^)	b.d	0.28 ± 0.145	0.65 ± 0.083	3.29 ± 1.088	n.d	n.d	0.78 ± 0.063	2.03 ± 0.205
${\text{PO}}_{4}^{3-}$ (μmol L^−1^)	0.10 ± 0.005	0.18 ± 0.005	0.12 ± 0.001	0.63 ± 0.005	0.01 ± 0.008	0.03 ± 0.026	0.02 ± 0.008	0.36 ± 0.032
Si(OH_4_) (μmol L^−1^)	0.70 ± 0.006	0.95 ± 0.016	0.40 ± 0.047	1.44 ± 0.040	0.16 ± 0.005	0.17 ± 0.002	0.18 ± 0.026	2.24 ± 0.102
Chl *a* (μg L^−1^)	2.8 ± 0.5	0.7 ± 0.2	2.8 ± 0.1	11.2 ± 0.4	0.2 ± 0.01	0.2 ± 0.01	0.2 ± 0.02	0.2 ± 0.04
Rel.abund. of diatoms (%)^c^	14	3	13	61	9	4	11	4

*n.d, not determined; b.d, below detection level.*

a *Potential temperature.*

b *Practical salinity.*

c *Relative abundance of diatoms revealed from the 18S rRNA gene amplification dataset*.

In the Nansen Basin, the DIC concentration was lowest in the samples collected directly under sea-ice (233.3 and 688.8 μmol kg^−1^), likely due to the dilution effect caused by sea-ice melt (Meire et al., 2015). In contrast, the highest DIC concentrations were obtained in the counterpart depth (20 m) at the same stations where the temperature was low and salinity was high. DOC concentration varied between stations (24.6–41.9 mg C L^−1^) and did not show a clear trend across the transect (Table 1). The overall inorganic nutrient concentration was higher in samples from NBm and NB2_20 than in other samples (Table 1). The *in vitro* chl *a* concentration was clearly highest (11.2 ±0.4 μg L^−1^) at the vicinity of the ice edge (NBm). In contrast, the lowest chl *a* concentrations were measured in the ice-covered Nansen Basin (Table 1).

### Taxonomic Components of Marine Microbiomes Sampled

**Figure 3:**
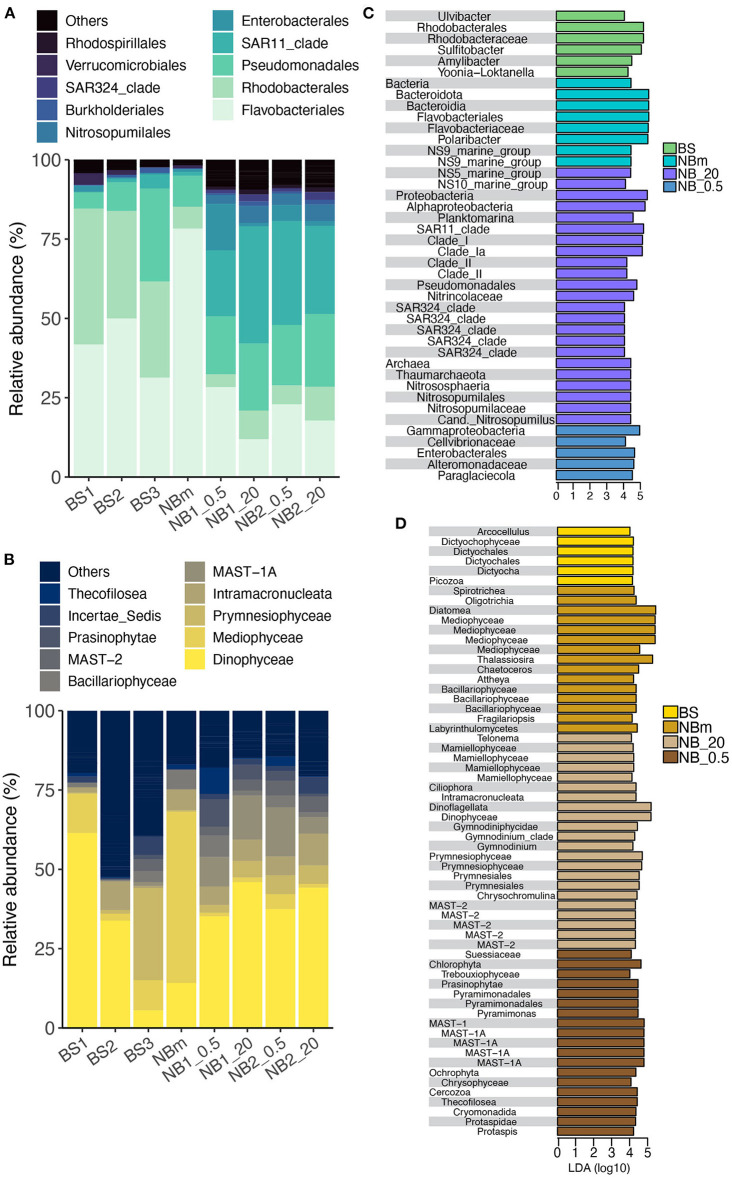
Comparison of prokaryotic and microeukaryotic members of the surface marine microbiomes sampled across the study's transect. The taxonomic profile of the most abundant prokaryotes **(A)** and microeukaryotes **(B)** retrieved from Amplicon Sequence Variants (ASVs) tables specified at the order and class taxonomic rank level, respectively. Prokaryotes **(C)** and microeukaryotes **(D)** identified from the LEfSe analysis at all taxonomic levels that describe the differences between each community grouped by environment type: BS, Barents Sea communities in open water environment; NBm, Nansen Basin margin community in sea-ice edge environment; NB_.5, Nansen Basin communities at the interface environment between sea-ice and surface water; NB_20, Nansen Basin communities in sea-ice covered environment. The x-axis is the LDA score (log 10) that indicates the scale of difference between groups.

The most common prokaryotic and microeukaryotic taxa (at the order and class level, respectively) were shared by all environments sampled, although relative abundances differed between locations (Figures 3A,B). The most common bacterial taxa were classified within the *Flavobacteriales* order (class *Bacteroidea*), which accounted for an average of 35% of the relative abundance of all 16S samples. However, an overwhelming predominance of ASVs classified as *Flavobacteriales* (relative abundance 78%) were observed at the NBm station, which represents both the boundary of Nansen Basin waters and the transition zone between ice-covered and open water environments (Figure 3A). The genus indicative of *Polaribacter* comprised the majority of ASVs belonging to the order *Flavobacteriales* at all stations with exception of the surface (20 m) Nansen Basin samples, where the genus NS5 marine group was predominant (Supplementary Data 1). The sampling stations located in the Barents Sea (BS1, BS2, and BS3) were also heavily comprised of sequences classified within the *Flavobacteriales* order (relative abundance 31–50%) but also composed of members within the orders of *Rhodobacterales* (relative abundance 30–43%; class *Alphaproteobacteria*) and *Pseudomonadales* (relative abundance 5–29%; class *Gammaproteobacteria*) (Figure 3A). The Nansen Basin stations (NB1 and NB2) also revealed the prevalence of ASVs indicative of the order *Pseudomonadales* (class *Gammaproteobacteria*), representing on average 20% of the relative abundances at these stations. The dominating members within *Pseudomonadales* were classified within the *Nitrincolacaea* family and genus SAR92 clade (Supplementary Data 1). In contrast, to open water and ice-edge communities, the heavily sea-ice-covered environment in the Nansen Basin was predominated by sequences identified within the order SAR11 (relative abundance 21–37%; class *Alphaproteobacteria*), including a majority of ASVs comprising the members of SAR11 clade Ia (Supplementary Data 1).

The most common microeukaryotic taxa, at the level of class, were indicative of *Dinophyceae*, which comprised an average of 35% of the relative abundance of all 18S samples (Figure 3B). However, there were two stations where the microeukaryotic portion of the microbiome was not predominated by ASVs belonging to the class *Dinophyceae*: BS3 in the Barents Sea and NBm at the vicinity of the ice-edge. The prevalent microeukaryotic ASVs at the NBm station were classified as class *Mediophyceae* (centric diatoms), which represented 54% of the relative abundance (Figure 3B) and consisted of sequences indicative of genus *Thalassiosira* (Supplementary Data 2). The NBm station is in the transition environment where the prokaryotic community was characterized by prokaryotic ASVs within the order *Flavobacteriales*. The relative abundance of *Dinophyceae* decreased along the Barents Sea toward the north (Figure 3B). The northernmost Barents Sea station, BS3, revealed a high relative abundance of sequences classified within the class *Prymnesiophyceae* (relative abundance of 30%), which were dominated by ASVs indicative of genera *Chrysochromulina* and *Phaeocystis* (Supplementary Data 2). In comparison to other stations, the Nansen Basin stations (NB1 and NB2) revealed higher relative abundances of ASVs indicative of organisms from within two MAST clades (marine stramenopiles): MAST-1A and MAST2 (Figure 3B).

The LEfSe method was used to infer indicator taxa that were differentially abundant among each grouped environment type (Segata et al., 2011). Relative abundances can show the predominant organisms in each environment, whereas LEfSe allows the detection of rare taxa that are significantly important per environment. LEfSe determines the significance of each taxonomic level and therefore also helps to infer whether only a certain genus is important or if the importance may be linked to higher taxonomic level similarities. It should be noted that results based on relative abundance metrics—such as LEfSe—can be highly time-dependent as the microbiome community structure tends to exhibit short-term temporal fluctuations in abundance (Needham and Fuhrman, 2016; Martin-Platero et al., 2018). The results show that multiple prokaryotic and microeukaryotic genera belonging to the same taxonomic order or class were differentially represented in different environments. LEfSe identified members of the order *Flavobacteriales* and more specifically the dominant member, *Polaribacter*, are indicator taxa of the ice-edge environment in the Nansen Basin margin (NBm), while the genus *Ulvibacter* is more prevalent in the open water Barents Sea (BS) at the time of this sampling campaign (Figure 3C). The sequences classified as genus *Planktomarina* belonging to the order *Rhodobacterales* represented indicator species of surface water in the sea-ice-covered Nansen Basin (NB_20). In comparison, three other genera from the same order (*Rhodobacterales*) were differentially represented as indicator taxa within the Barents Sea community (BS). The Nansen Basin (NB_20) was distinguished from other environments by the prevalence of organisms within the kingdom *Archaea*, which was represented by members indicative of the family *Nitrospumilaceae*. The microeukaryote communities in the Nansen Basin (NB_0.5 and NB_20) were nearly solely described by nanophytoplanktons and picophytoplanktons, such as classes *Prasinophytae* and *Mamiellophyceae* from the phylum *Chlorophyta*, and MAST-1A and MAST-2 clades and small dinoflagellates (families *Gymnodinium* clade and *Successiae*) (Figure 3D).

### Diversity and Overlapping Distribution of Taxa Across Environments

**Figure 4:**
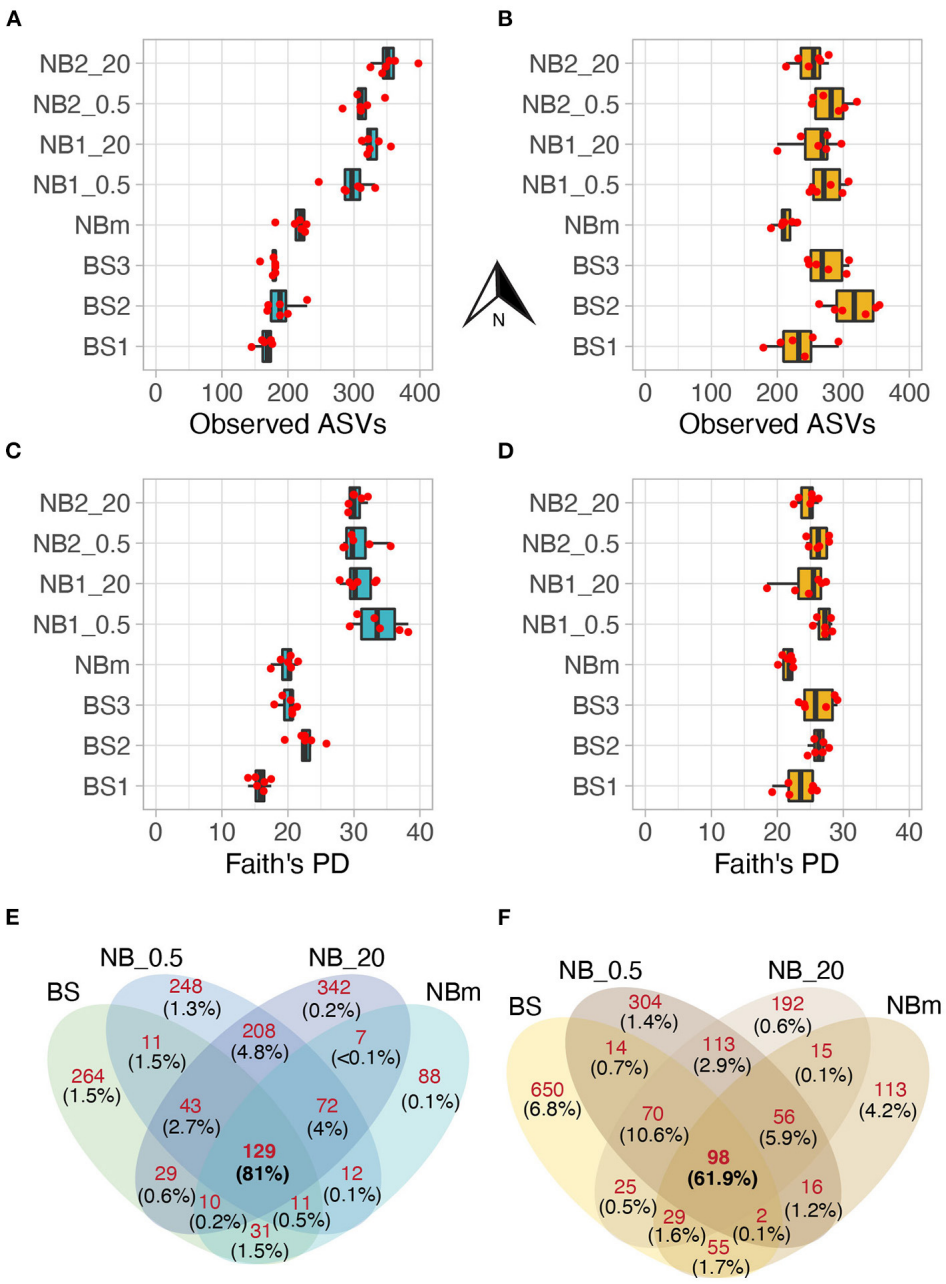
The prokaryotic **(A)** and microeukaryotic **(B)** richness were measured as the observed number of ASVs. Corresponding Faith's Phylogenetic Diversity (PD) values for prokaryotes **(C)** and microeukaryotes **(D)**. The values of biological replicates (*n* = 6) are shown over boxplots, and the arrow denotes a northward direction of sample stations. Alpha diversity showed a distinct increasing trend along the study transect for the prokaryotic but not the eukaryotic members of the sampled microbiomes. The diversity increases with increasing Faith's PD. Venn diagrams reporting a number of exclusive prokaryotic **(E)** and microeukaryotic **(F)** ASVs between grouped communities. Integers (shown in red color) represent the shared/exclusive number of ASVs. The relative abundance as a proportion of sequences of these ASVs from a total number of sequences is shown in parenthesis (black color). The ASVs shared by all grouped communities comprised the majority of total sequences per 16S and 18S data set. For example, **(F)** 129 ASVs shared by all grouped communities have high relative abundance as these ASVs comprised 81% of all sequences in the 16S dataset, whereas 264 ASVs exclusive to BS stations accounted for only 1.5% of a total number of sequences in the same dataset.

The observed number of prokaryotic ASVs increased from the south (ASVs: 166 ± 12) toward the north (ASVs: 355 ± 24) and this increase was statistically significant (Tukey, *p* < 0.05) (Figure 4A; Supplementary Table 2), while the microeukaryotic richness had no significant differences between the north and south stations (Figure 4B; Supplementary Table 2). The prokaryotic diversity (Faith's PD) was statistically significant (Tukey, *p* < 0.05) (Supplementary Table 3) and increased from the south (PD: 16 ± 1.2) toward the north (PD: 30 ± 1.2) (Figure 4C), while this was not observed for the microeukaryotes (Figure 4D; Supplementary Table 3).

The total number of classified prokaryotic and microeukaryote ASVs after rarefaction were 1505 and 1752, respectively, from which a considerably low number of ASVs were shared by all grouped communities (Figures 4E,F). However, the ASVs that were shared between all environment groups were highly abundant in their relative abundances as these ASVs comprised 81% and 61.9% of the total prokaryotic and microeukaryote sequences, respectively. In contrast, the high number of observed ASVs that were exclusive to each environment type accounted for the low proportion of the total sequences (Figures 4E,F).

### Biological and Physical Factors Are Associated With Different Microbiome Structures

**Figure 5:**
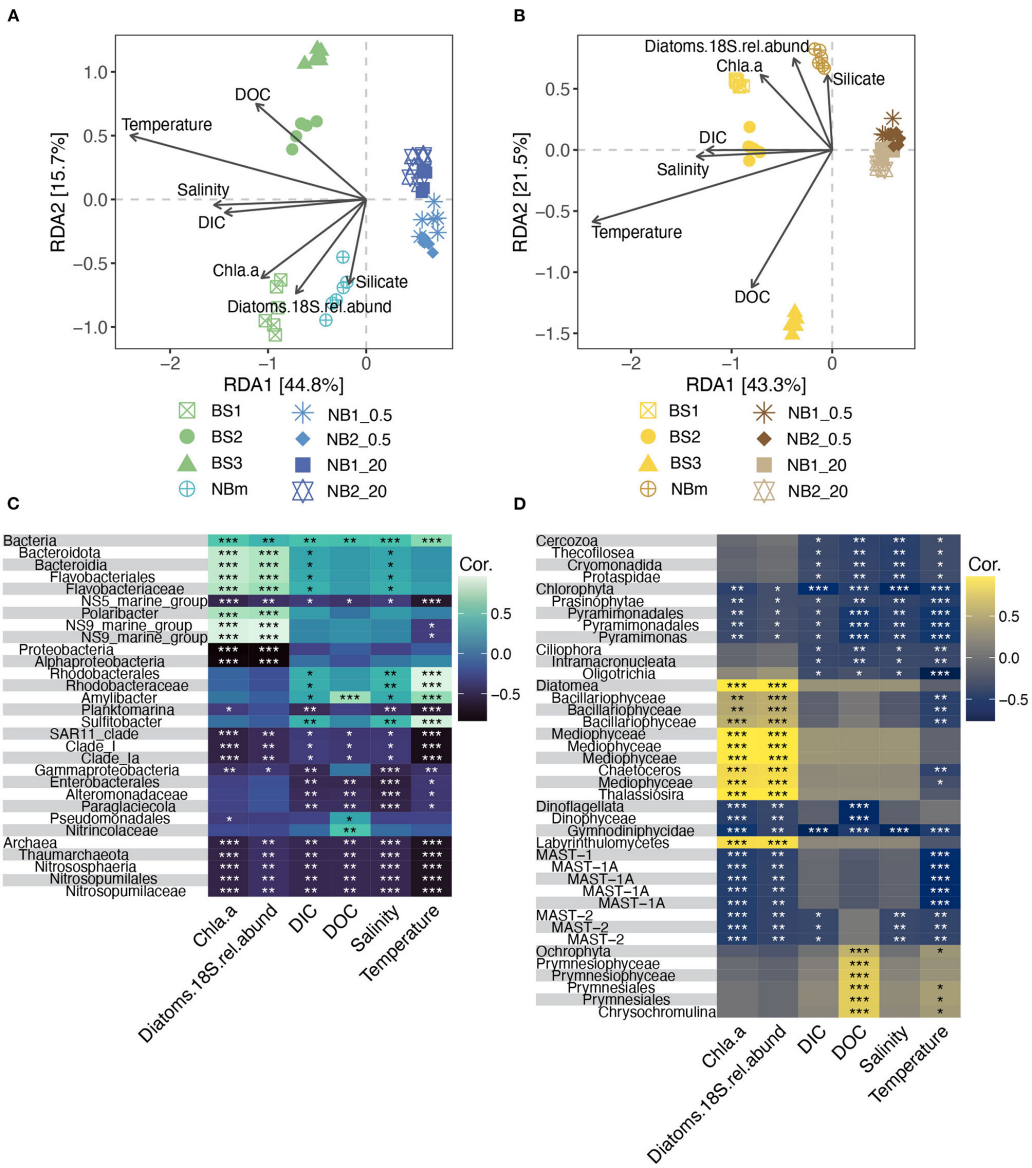
Beta diversity and the contextual environmental measurements showed similar patterns of differences for both prokaryotic and microeukaryotic communities compared between all sampling stations. Distance-based Redundancy analysis (dbRDA) biplot of prokaryotic **(A)** and microeukaryotic **(B)** beta diversity measured as unweighted UniFrac distances (sample scores) and environmental factors as arrows pointing to the direction of maximum variation of the respective factor. Heatmaps showing Pearson's correlation between environmental factors and differentially represented prokaryotic **(C)** and microeukaryotic **(D)** taxa were obtained from the LEfSe analysis. The color indicates Pearson's correlation coefficient according to the color legend and the level of significance is marked with * (*, *p* ≤ 0.05; **, *p* ≤ 0.01; ***, *p* ≤ 0.001).

The observable differences in community composition between samples were also represented by different environmental measurements. Beta diversity was visualized *via* the dbRDA analysis showing significant dissimilarities in prokaryotic (PERMANOVA, *r*^2^ = 0.71, *p* = 0.001) and microeukaryotic (PERMANOVA, *r*^2^ = 0.78, *p* = 0.001) community composition across all samples (Figures 5A,B). The prokaryotic and eukaryotic components of each environmentally grouped microbiome showed similar dissimilarity patterns. The dbRDA analysis revealed that the environmental factors explained in total (sum of RDA1 and RDA2) 60.5 and 64.8% of the variance among prokaryotes and microeukaryotes, respectively (Figures 5A,B). The first RDA component indicates the variation of communities between the Barents Sea, the Nansen Basin margin, and the Nansen Basin for both prokaryotic and microeukaryotic ASVs. The environmental factors salinity, DIC, and temperature correlated with the first RDA component and may drive the differences in community compositions between these regions (Figures 5A,B). As salinity and DIC showed a strong correlation with each other (DIC concentration is dependent on salinity), it is difficult to separate the influence of these two factors on community composition. This correlation is presumably highly influenced by very low salinity under sea-ice bottom as the DIC concentration was higher in Nansen Basin at 20 m (NB1_20 and NB2_20) than in Barents Sea samples (BS1, BS2, and BS3) with higher salinity (Table 1). Also, chl *a* and relative abundance of diatoms are interrelated, but due to the compositional nature of diatoms, these measurements explain the environment differently, e.g., the relative abundance of diatoms can be similar between samples but chl *a* concentration can be highly different as shown in Table 1. The variation between Barents Sea stations, as well as among them (BS1, BS2, and BS3), and NBm station was explained mainly by the second RDA component, which accounted for 15.7 and 21.5% of the variance in prokaryotic and microeukaryotic community composition, respectively (Figures 5A,B). The concentration of silicate and relative abundance of diatoms and to a lesser extent chl *a* and DOC were the environmental factors that correlated more with the second RDA component and may explain the observed differences in microbial community compositions between the stations in the Barents Sea (BS1, BS2, and BS3) and the Nansen Basin margin (NBm) (Figures 5A,B).

The role that specific environmental factors play in structuring each unique microbiome was also inferred from Pearson's correlations between the environmental measurements and prokaryotic and eukaryotic taxa obtained from the LEfSe analysis (Figures 5C,D). Most of the differentially abundant indicator taxa (Figures 3C,D) for the Barents Sea (BS) and the Nansen Basin margin (NBm) showed positive correlations to the environment factors (Figures 5C,D). This trend was opposite for differentially represented taxa (indicator taxa) identified for the heavily sea-ice-covered Nansen Basin (Figures 3C,D, 5C,D). The weaker negative correlation between prokaryotic and microeukaryotic indicator taxa for NB_20 (Nansen Basin 20 m samples) and relative abundance of diatoms than chl *a* is explained by higher diatom abundance compared to under sea-ice bottom samples (NB_0.5) (Table 1, Figures 3C,D, 5C,D). The sequences classified as *Sulfitobacter* and *Amylibacter* within the order *Rhodobacterales* were indicator taxa for the Barents Sea (Figure 3C) and showed significant (positive) relationships to temperature, salinity, and DIC (Table 1; Figure 5C). Instead, ASVs indicative of members from the order *Flavobacteriales*, except the NS5 marine group, were the predominant taxa at the NBm station (Figure 3C) and showed strong positive (significant) relationships to chl *a* and the relative abundance of diatoms, but not temperature (Figures 5A,B). This is likely because the order *Flavobacteriales* was also found to be relatively abundant within the colder Nansen Basin environments (Figure 3A). The microeukaryotic taxa that were prevalent in both the Barents Sea and the Nansen Basin were indicative of members from within the class *Prymnesiophyceae* (Supplementary Data 3) but showed no significant correlations to most of the environment factors (Figure 5D). Weak correlations were also observed within some of the differentially abundant indicator taxa from the Nansen Basin margin (i.e., class *Mediophyceae*) (Figures 3D, 5D). Many of the indicator taxa from the low-salinity lens at the sea-to-ice interface environment in rge Nansen Basin (NB_0.5), such as ASVs indicative of phyla *Chlorophyta* and *Cercozoa*, showed strong negative correlations to salinity (Figures 3D, 5D).

### Phylogenetic Diversity and Inference of Selection Processes Between Environments

**Figure 6:**
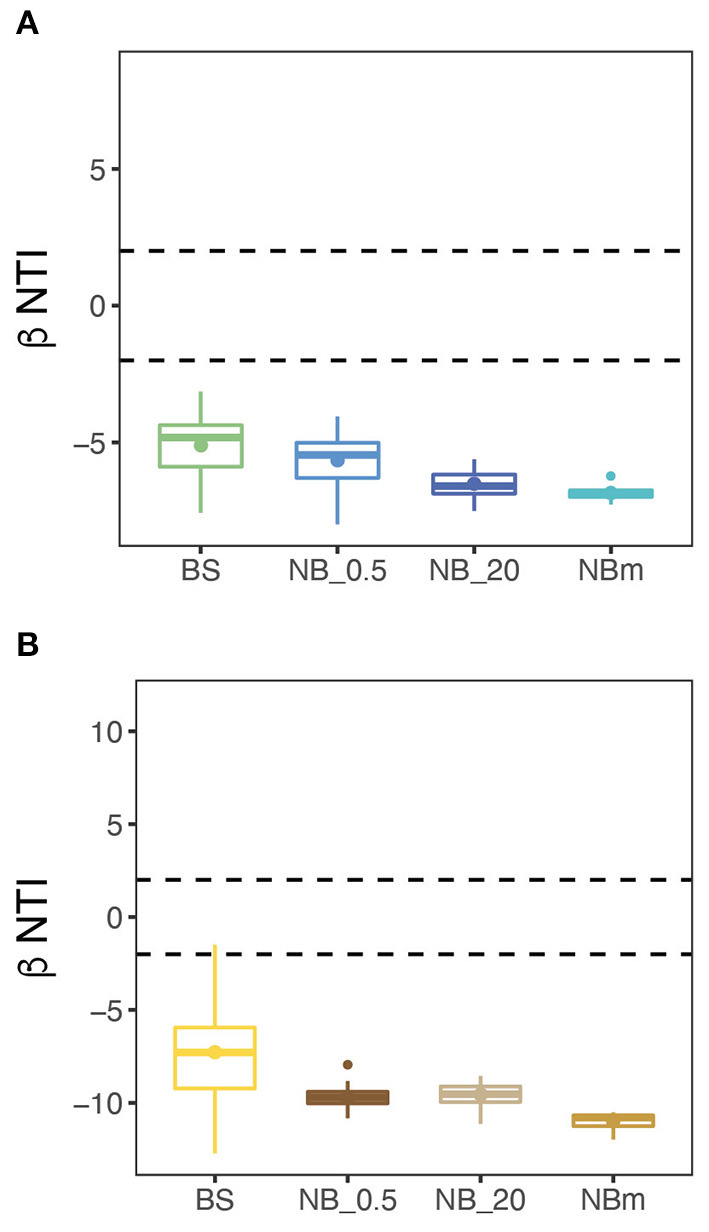
Null model analysis suggests that a strong deterministic selection process with homogeneous selection is prevalent across the study transect. This was supported by the Beta Nearest Taxon Index (βNTI) for unweighted abundances between grouped prokaryote **(A)** and microeukaryote **(B)** communities indicating strong consistent (homogeneous) community selection. Dashed lines mark the significance thresholds (βNTI > 2 and βNTI < −2) for deterministic selection processes: βNTI > 2 is an indication for variable deterministic selection and βNTI < −2 indicates homogeneous deterministic selection. The stochastic selection process occurs when βNTI-values are between the dashed lines (−2 < βNTI < 2).

The investigation of ecological processes governing microbial community composition revealed that the community composition between grouped communities was more similar than expected by randomization (Stegen et al., 2012). This was determined from null model analysis by calculating βNTI values both for prokaryote and eukaryote communities. The βNTI values were clearly below the significance threshold (-2), thus indicating strong homogeneous deterministic selection with consistent selection pressure across the entire sampling transect (Figure 6). This means that environmental factors are likely select for the observed phylogenetic composition. This result also suggests that the community members are phylogenetically spread, i.e., more distantly related than expected by chance. The calculated Faith's phylogenetic diversity (PD) values also support this inference, where the relative level of phylogenetic clustering or paucity of clustering was observed to be similar between communities as the values increased with richness (Figures 4C,D).

## Discussion

This study characterized and compared the structure and selection of surface marine microbiomes across specific environments spanning the Atlantic-influenced Barents Sea to the heavily sea-ice-covered Nansen Basin waters. We found that the most common microeukaryotic and prokaryotic taxa were shared across the sampling locations and that these taxa were similar to what has been observed previously in the Nansen Basin, as well as more broadly in the Arctic Ocean (Hatam et al., 2014; Rapp et al., 2018; Signori et al., 2018; de Sousa et al., 2019). Here, the prokaryotic members of each community showed a clear increase in richness and diversity (i.e., Faith's PD) across the transect from south to north (Figure 4C). This increase occurred alongside a decrease in surface temperature and the transition from the open water to the sea-ice dominating environment.

Our findings on diversity trends over the northward transect are in contrast with the current understanding of latitudinal decrease in upper water microbial richness and diversity due to changes in temperature, which has been reported previously (Fuhrman et al., 2008; Ibarbalz et al., 2019; Martin et al., 2021). Our findings can be partly explained by the recent empirical-based demonstration of temperature breakpoints, 9.49°C for prokaryotes and 13.96°C for eukaryotes, which separate cold microbial communities (determined from beta-diversity) and their associated metabolism from warm ones (Martin et al., 2021). This means that further temperature decreases below these recently suggested ecological breakpoints do not necessarily negatively impact microbial diversity (Supplementary Figure 5), as we have observed here. The cold temperature may be a major selection force for community composition but the separation to “cold” microbiomes likely occurs further south of this studied area (Martin et al., 2021). The temperature range of this relatively large study area across the Arctic Ocean (-1.8 − +6.9°C) can therefore be considered as a relatively homogeneous and consistent habitat for the microorganisms representing certain parts of the phylogeny that have been selected by cold temperature as a strong environmental filter (Vellend, 2010; Dini-Andreote et al., 2015). This is supported by our results from βNTI null models (Figure 6) that inferred strong deterministic homogeneous selection governing more similar microbial taxa pools than expected by chance (Stegen et al., 2012; Brislawn et al., 2019). Consequently, the observed difference in relative abundances of the members of this pool is instead determined mainly by other factors than temperature. The results obtained from this study are limited to single snapshots at certain times taken across a large geographical area of the Arctic Ocean and certainly cannot account for all ecological niches resident in the region throughout the year. Therefore, we do not attempt to project these results to infer phylogenetic selection processes that may be occurring during fall, winter, or spring conditions.

In addition to temperature as a prevailing selective force, the constant northward traveling Atlantic currents in the area of the Arctic Ocean studied here may also contribute to continuous habitat formation. This may enable some level of horizontal species dispersion, which is necessary; otherwise, stochasticity is more likely to become the prevalent ecological process governing community composition between geographical locations (Evans et al., 2017). Although the surface waters in the Nansen Basin margin and the Nansen Basin were characterized by Polar Surface water (Figure 2), the presence of modified Atlantic water has been observed in the Nansen Basin above 100 m (Meyer et al., 2017). Thus, there are no physical limitations for the surface water microbial taxa pool that have passed the temperature filter to take advantage of the entire region.

Multiple studies have addressed the present and future impact of Atlantification on microbiomes in the North Atlantic, the Barents Sea, and to the west and north of Svalbard. Many of these recent observations and future predictions have focused on shifts in phytoplankton biogeography (poleward expansion), the blooming dynamics, and/or the relative abundances of phytoplankton rather than the distinction of specific taxa or notably the invasion of new taxa (Barton et al., 2016; Carter-Gates et al., 2020; Orkney et al., 2020; Oziel et al., 2020; Cardozo-Mino et al., 2021). These changes were mainly connected to changing bottom-up controls and intensified surface current velocities instead rather than being directly linked to increasing temperature. It is less clear how shifts in prokaryote components of these microbiomes might be restructured by Atlantification. It has been proposed that Atlantification may cause the extinction of rare microbial taxa that are more localized and niche specialized and thus have a lower tolerance to environmental changes compared to the abundant taxa (Carter-Gates et al., 2020). We did not emphasize the rare taxa in this study although a high number of rare ASVs were observed between different environment types (Figures 4E,F). According to the model-predicted future temperature regimes in the North Sea, the temperature is expected to rise, although not above the recently identified temperature-dependent diversity breakpoints (Martin et al., 2021). Therefore, it is unlikely that the surface water-associated microbial taxa, especially the most abundant members, will change dramatically across the investigated area in the near future. In turn, the stronger presence of Atlantic water may even act as a stronger homogeneous selection between the Barents Sea and the Nansen Basin.

One of the goals of this study was to determine if and how microbiomes inhabiting waters directly under the sea ice are different from those sampled from the surface waters at 20 m. We found a high degree of similarity in microbial communities between the two sampled depths (0.5 m and 20 m) in the Nansen Basin (Figures 5A,B), which highlights that vertical mixing likely plays a larger role in prokaryotic and microeukaryotic community composition than environmental selection with respect to differences in temperature and salinity. This insight is supported by previous findings that revealed high vertical similarity in microbial community structure between two surface depths occurring above the MLD both in sea-ice-covered and open water areas (de Sousa et al., 2019; Allen et al., 2020). However, in this study, we detected a strong difference in salinity between the depths whereas the abiotic environment was relatively constant in the previous studies that showed similar findings. It is possible that the microbial community in the low-salinity layer under the sea ice can become partly isolated (trapped) during the progressive sea-ice melting, which can potentially lead to a variation in the selective environment (Dini-Andreote et al., 2015). However, without measurements that directly assess the vertical flux of cells between 20 m and the sea-ice interface, it is difficult to estimate how strongly the organisms in the low-salinity layer were isolated from the community below or the temporal scale to evaluate the development of this layer. Since the pycnocline was very sharp, it suggests that the physical mixing and thus community blending across the halocline was at least temporally limited.

We observed differentially represented taxa in the Nansen Basin between the different surface water depths (0.5 m and 20 m) (Figures 3C,D), yet the results did not reveal clear evidence of whether the change in salinity would favor certain taxa over the others among the “trapped” taxa pool. Among the indicator microeukaryote taxa for the Nansen Basin (NB_0.5 and NB_20), a majority were classified as taxa known to be heterotrophic or mixotrophic. These microeukaryotes are widely distributed across high-latitudes and/or global waters comprising organisms indicative of members within the class *Intramacronucleata* (Ciliates), phylum *Cercozoa*, orders MAST-1A and MAST*-*2, and Gymnodinium-like dinoflagellates from the order *Gymnodiniphycidae*. These taxa are often found in low-irradiance and low-nutrient environments that are prevalent under heavy sea ice (such as the Nansen Basin) (Levinsen et al., 2000; Rapp et al., 2018). Hence, they do not necessarily represent specificity for Polar Surface water or brackish systems. Contrastingly, these taxa, with the exception of MAST-1A, revealed a strong negative relationship to salinity. In the Nansen Basin, the differentially represented ASVs indicative of class *Mamiellophyceae* for 20 m and classes *Prasinophyceae* and *Trebouxiophyceae* for 0.5 m, all members of the phylum *Chlorophyta*, are highly common photoautotrophs in global coastal waters and they have a wide distribution with respect to temperature and salinity (Tragin and Vaulot, 2018). *Pyramimonas*, a genus within the *Prasinophyceae* class, can dominate the high Arctic near-surface picophytoplankton community under and between sea-ice floes in summer (Zhang et al., 2015), which is compatible with the presented results of the sea-to-ice interface community of the Nansen Basin (Figure 3D). Also, the predominance of class *Dinophyceae* in Nansen Basin, as well as, within some of the Barents Sea stations (BS1 and BS2) is not uncommon as its strong presence is well documented in both areas (Wassmann et al., 2006a; Rapp et al., 2018; de Sousa et al., 2019).

The phylum *Thaumarchaeota* (kingdom *Archaea*) contains mainly members of ammonium oxidizers like Candidatus Nitrosopumilus, which was the only thaumarchaeal genus observed here, (Kirchman et al., 2007; Connelly et al., 2014), was nearly exclusively present at the Nansen Basin stations (NB1 and NB2) and differentially abundant in 20 m (NB_20) (Figures 3C,D), although in lower abundance than what was observed in winter surface waters in the Nansen Basin (de Sousa et al., 2019). The explanation for this is likely not related to salinity but to the impact of sea-ice on light availability in the ocean water below, as most of the ammonia-oxidizing archaea are known to be light-sensitive and inhibited by harmful reactive oxygen species produced by increased irradiance (Tolar et al., 2016). This may explain the near absence of *Thaumarcheota* in Barents Sea surface waters as the members of this phylum are often found abundant in the mesopelagic layer (Karner et al., 2001).

Diatoms are prevalent in surface waters and sea-ice-associated marine microbiomes with an important role in the Arctic marine ecosystem functioning by influencing biogeochemical cycles, transporting organic matter to higher trophic levels, and contributing to biological carbon sequestration (Wassmann et al., 2006a; Litchman et al., 2015; Tréguer et al., 2018). As these processes are also aided by interacting microbes, we sought to better understand how diatoms might structure localized communities across the Barents Sea and the Nansen Basin by evaluating the bacterial response on phytoplankton blooms observed between sampling locations. Large diatom blooms are often associated with higher DOC concentrations (Norrman et al., 1995; Smith et al., 1995), yet DOC was relatively similar across the study area (Table 1), which further supports an inference of more homogeneous selection across larger geographical areas than initially suspected. Although the DOC compound composition was likely variable due to different sources, variable substrate availability might promote the paucity of phylogenetic clustering as several taxa are known to have the ability to not only utilize a wide spectrum of organic matter but also have different preferences between taxa within the same group (Teeling et al., 2016; Underwood et al., 2019). Our study lacks the temporal scale required to detect diatom “bloom” events, yet we detected spatial variation in chl *a* and relative abundance of diatoms as well as in the relative abundances of the most common microeukaryote taxa. Thus, more comprehensive, diatom bloom targeted studies are needed to fully describe the microbiome succession in detail. We concluded that the Barents Sea stations (BS1, BS2, and BS3) exhibited phytoplankton post-bloom conditions at the time that it was sampled for this study and presumably at some different stages between stations based on the measured chl *a* concentration, the eukaryotic community composition, and the previously documented timing of phytoplankton bloom from that area (Wassmann et al., 2006a; Oziel et al., 2017). However, the high chl *a* concentration (11.2 μg L^−1^) and proportional abundance of centric diatoms (i.e., genus *Thalassiosira*) observed at NBm station revealed a strong indication of prevailing ice-edge bloom and intense primary production (Wassmann et al., 2006a). Very low chl concentrations were measured at the Nansen Basin stations (NB1 and NB2), and thus, these stations were considered to have low autotrophic phytoplankton biomass. Also, the higher DIC concentration at 20 m than at the other stations indicated lower inorganic carbon uptake (Norrman et al., 1995; Meire et al., 2015).

The Barents Sea and the Nansen Basin margins were dominated by ASVs indicative of copiotrophic bacteria like the predominant genus *Polaribacter* (order *Flavobacteriales*) and order *Rhodobacterales*, which are attributed to phytoplankton blooms as they are known to utilize phytoplankton-derived exudates (Teeling et al., 2012; Buchan et al., 2014). The significantly high contribution of *Polaribacter* to the community in the ice-edge at the NBm station can therefore be connected to the exemplary centric diatom thriven surface water ice-edge bloom. The differentially abundant order *Rhodobacterales* and the predominant genera (*Sulfitobacter* and *Amylibacter*) in the Barents Sea were the only prokaryotic taxa that showed a significant positive correlation with temperature (Figure 5C), corresponding to the previous finding of *Sulfitobacter* in relatively warm, saline, and productive Arctic surface water (Lee et al., 2019). However, *Sulfitobacter* has also been found in association with diatom-dominated ice-algal aggregates in the high Arctic Ocean (Rapp et al., 2018), suggesting that its very low presence in the Nansen Basin was not temperature-limited but instead due to low algal biomass. Many Rhodobacteria affiliates are rapid algal colonizers (Buchan et al., 2014), and therefore, their low relative abundance in the Nansen Basin margin was not expected. It is possible that the initial abundance of *Polaribacter* was already relatively high prior to the ice-edge bloom as it can be also abundant in sea-ice-associated communities (Rapp et al., 2018) and therefore responded faster to the inferred bloom than *Sulfitobacter*. The high relative abundance of *Rhodobacterales* at the Barents Sea stations (BS1, BS2, and BS3) may be related to the decline of *Polaribacter* in post-bloom conditions with the decrease of diatom abundance (Teeling et al., 2012).

The Nansen Basin was characterized by low phytoplankton biomass (as a proxy from chl *a*) and the prokaryotic community was dominated by taxa that have been classified as small molecule degraders such as SAR11 clade (class *Alphaproteobacteria*) and *Pseudomonadales* (class *Gammaproteobacteria*) (Giovannoni, 2017; Francis et al., 2021). Although sequences classified within the order *Flavobacteriales*, notably genera *Polaribacter* and NS5 marine group, were also abundant in the Nansen Basin, these two genera have been observed in the Arctic under sea-ice before (Rapp et al., 2018) and *Polaribacter* is known to utilize organic matter from variable sources (Teeling et al., 2012; Underwood et al., 2019). The high relative abundance of SAR11 clade Ia (class *Alphaproteobacteria*) observed at NB1 and NB2 stations corresponded to the findings in epipelagic waters between the Yermak Plateau and the southern Nansen Basin (de Sousa et al., 2019). The SAR11 clade is ubiquitous, and it is suggested to be one of the most abundant bacterioplankton in surface oceans prospering oligotrophic systems both in dark and light conditions (Morris et al., 2002) using mainly small organic molecules (Gomez-Pereira et al., 2013). Also, SAR11 can utilize exogenous sources of sulfur-like dimethylsulfoniopropionate (DMSP) (Tripp et al., 2008). The high relative abundance of both SAR11 clade and dinoflagellates in the sea-ice-covered Nansen Basin in this study (Figure 3) corresponds with a previous finding from the same area, where it was suggested that the growth of SAR11 under sea-ice may be sustained by DMSP produced by dinoflagellates (de Sousa et al., 2019). SAR11 clade was also present at other stations although in low relative abundance, likely due to being outcompeted by faster-growing copiotrophs (Teeling et al., 2012).

## Conclusion

This study investigated the phylogenetic diversity and structure of the cold marine ecosystems across different environments and specifically drew comparisons between the Atlantic-influenced Barents Sea and Nansen Basin environments. A sampling of open water in the Barents Sea and alongside a sea-ice edge in the Nansen Basin margin revealed the dominance of copiotrophic prokaryote taxa such as genera *Polaribacter* and *Sulfitobacter*, which are thought to be associated with phytoplankton bloom/post-bloom phenology and the resultant high abundance of diatoms. Whereas, highly sea-ice-covered environments in the high Arctic Nansen Basin were more characterized by widely common nanophytoplankton and picophytoplankton and oligotrophic prokaryotes, like SAR11 clade. In accordance with previous findings, we surmise that phytoplankton blooms are an important factor in determining the bacterial community structure in Arctic surface waters. Our results suggest that both prokaryotic and microeukaryotic components of the surface marine microbiome were subjected to deterministic homogeneous selection, which is an indication of the presence of strong environmental selection that was consistent across the large area of the Arctic Ocean surveyed here. Furthermore, we found that the decreasing temperature along the sampling transect did not negatively impact microbial species richness. This is likely due to temperature-driven selection for cold- and warm-water microbiomes that takes effect at warmer temperatures than are present in the contemporary Barent Sea environment. Hence, despite the major influence of Atlantic waters, this selection occurs further south (in the North Sea) where the temperature-dependent microbial diversity breakpoints have been observed (Martin et al., 2021). We also suggest that the influence of Atlantic water between the Barents Sea (strongly influenced) and the Nansen Basin (less influenced) has implications for homogeneous habitat formation by allowing a necessary level of horizontal species dispersion. Although this study covered a large geographical area, the spatial sampling resolution is small in comparison to larger studies such as the Tara Oceans project. However, this study presents research questions focused on the Barents Sea and the Nansen Basin, which are difficult to access and therefore underrepresented in marine microbiology. We believe long-term time-series studies across this transect are needed to infer the real-time role of Atlantification on microbial community selection. The null modeling approach can be considered a valuable tool for that, as it helps to identify the role of ecological selection resulting from the Atlantification of marine microbiomes.

## Data Availability Statement

The datasets presented in this study can be found in online repositories: https://osf.io/g8wxc/.

## Author Contributions

NJA and HCB conceptualized the study and conducted data analyses. NJA, HDS, and HCB conducted the fieldwork. NJA and KC processed the environmental samples. HDS and SK processed molecular samples and amplicon sequence analyses. NJA wrote the manuscript draft. All authors contributed to the final version.

## Funding

This work was supported by strategic funding allocation, from UiT—The Arctic University of Norway to the project ABSORB—Arctic Carbon Storage from Biomes.

## Conflict of Interest

The authors declare that the research was conducted in the absence of any commercial or financial relationships that could be construed as a potential conflict of interest.

## Publisher's Note

All claims expressed in this article are solely those of the authors and do not necessarily represent those of their affiliated organizations, or those of the publisher, the editors and the reviewers. Any product that may be evaluated in this article, or claim that may be made by its manufacturer, is not guaranteed or endorsed by the publisher.
