# Supplementary material for: Diversity and Selection of Surface Marine Microbiomes in the Atlantic-Influenced Arctic
Source: Front Microbiol. 2022 Jul 14;13:892634. doi: 10.3389/fmicb.2022.892634 (PMC9329088; doi:10.3389/fmicb.2022.892634)
Supplement: Supplementary file 1 [file Data_Sheet_1.zip › Datasheet 1/Supplementary Material.pdf]

SUPPLEMENTARY FIGURES AND TABLES

Diversity and selection of surface marine microbiomes in the Atlantic-influenced Arctic

Nerea J. Aalto[1,2], Hannah Schweizer[1,2], Stina Krsmanovic[1], Karley Campbell[1,3], Hans C. Bernstein[1,2]*

[1]Faculty of Biosciences, Fisheries and Economics, UiT - The Arctic University of Norway, 9019, Tromsø, Norway; [2]The Arctic Centre for Sustainable Energy, UiT - The Arctic University of Norway, 9019, Tromsø, Norway; [3]Bristol Glaciology Centre, University of Bristol, United Kingdom

* ***Correspondence:*** Hans C. Bernstein, UiT – The Arctic University of Norway, BFE-fak., Postboks 6050, Langnes, 9037 Tromsø, Norway, Email: hans.c.bernstein@uit.no

Supplementary Table S1. Sampling dates and station locations.

Location	Station	Position (decimal degrees)	Sampling date
Barents Sea	BS1	71.7 N, 26.7 E	04.07.2019
Barents Sea	BS2	74.0 N, 27.2 E	05.07.2019
Barents Sea	BS3	75.5 N, 27.7 E	06.07.2019
Nansen Basin margin	NBm	81.3 N, 19.8 E	18.07.2019
Nansen Basin	NB1	84.2 N, 29.1 E	11.07.2019
Nansen Basin	NB2	84.7 N, 28.8 E	13.07.2019

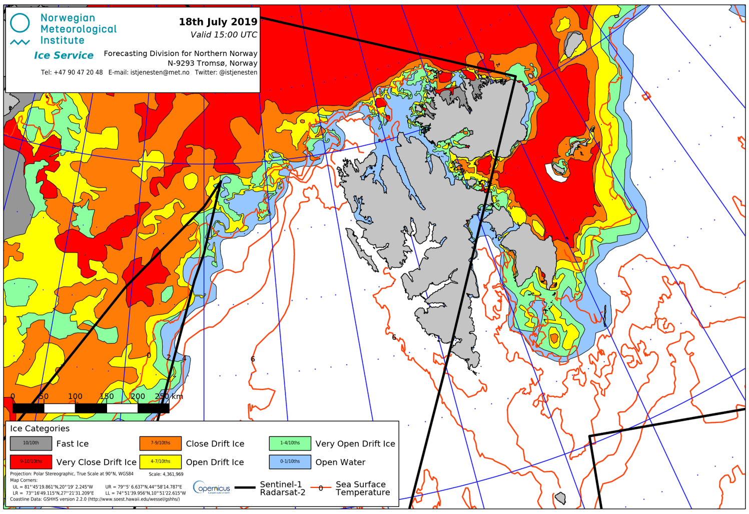

Supplementary Figure S1. Sea-ice cover around Svalbard at the time of sampling in the vicinity of ice-edge station, NBm. The figure is obtained from the public online service of the Norwegian Meteorological Institute (MET).

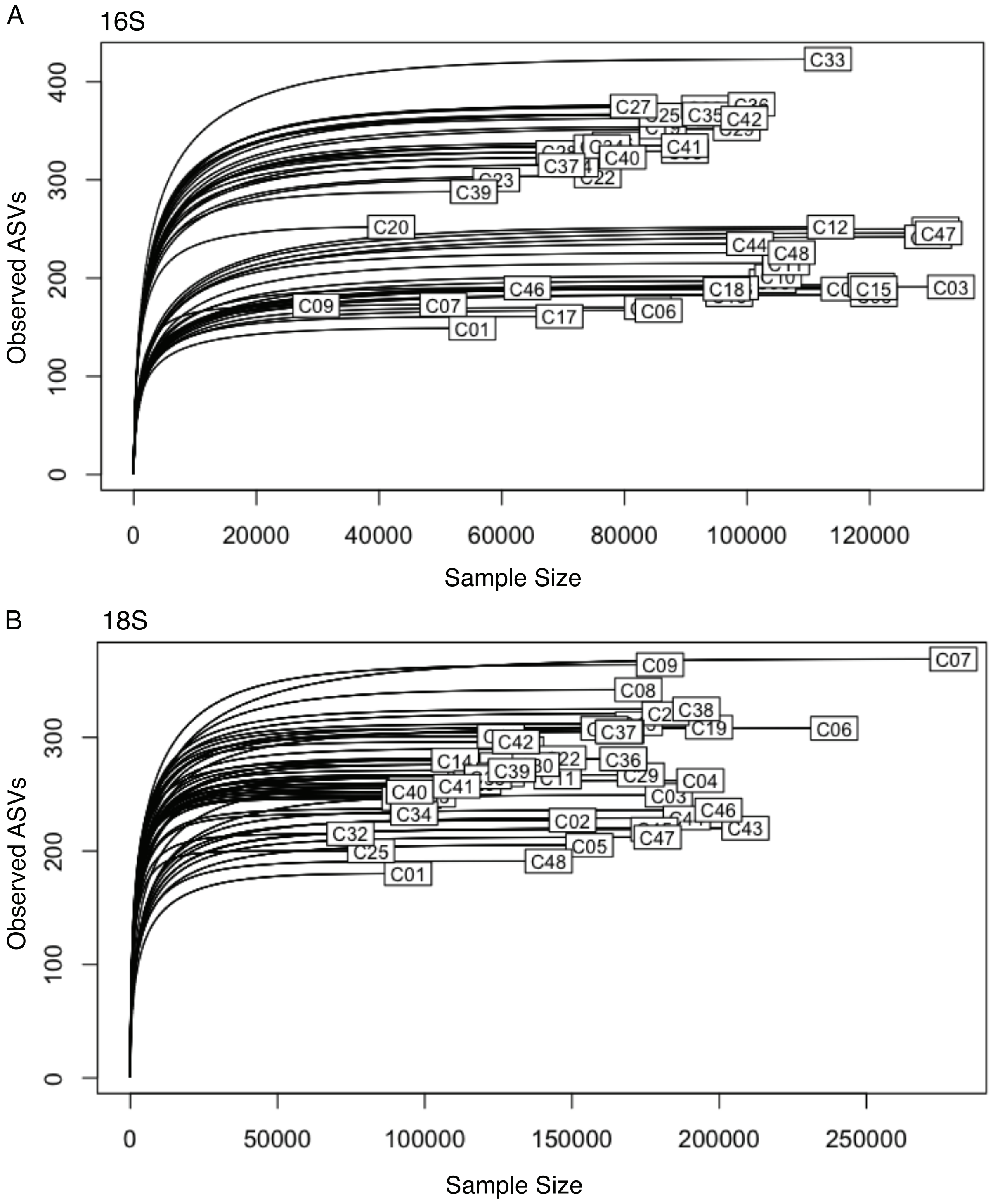

Supplementary Figure S2. Rarefaction curves showing observed number of ASVs and sequencing depth of each sequenced sample in A) prokaryotic (16S) and (B) microeukaryotic (18S) data sets. The minimum number of sequences – 29746 for 16S and 74750 for 18S – was used for rarefaction.

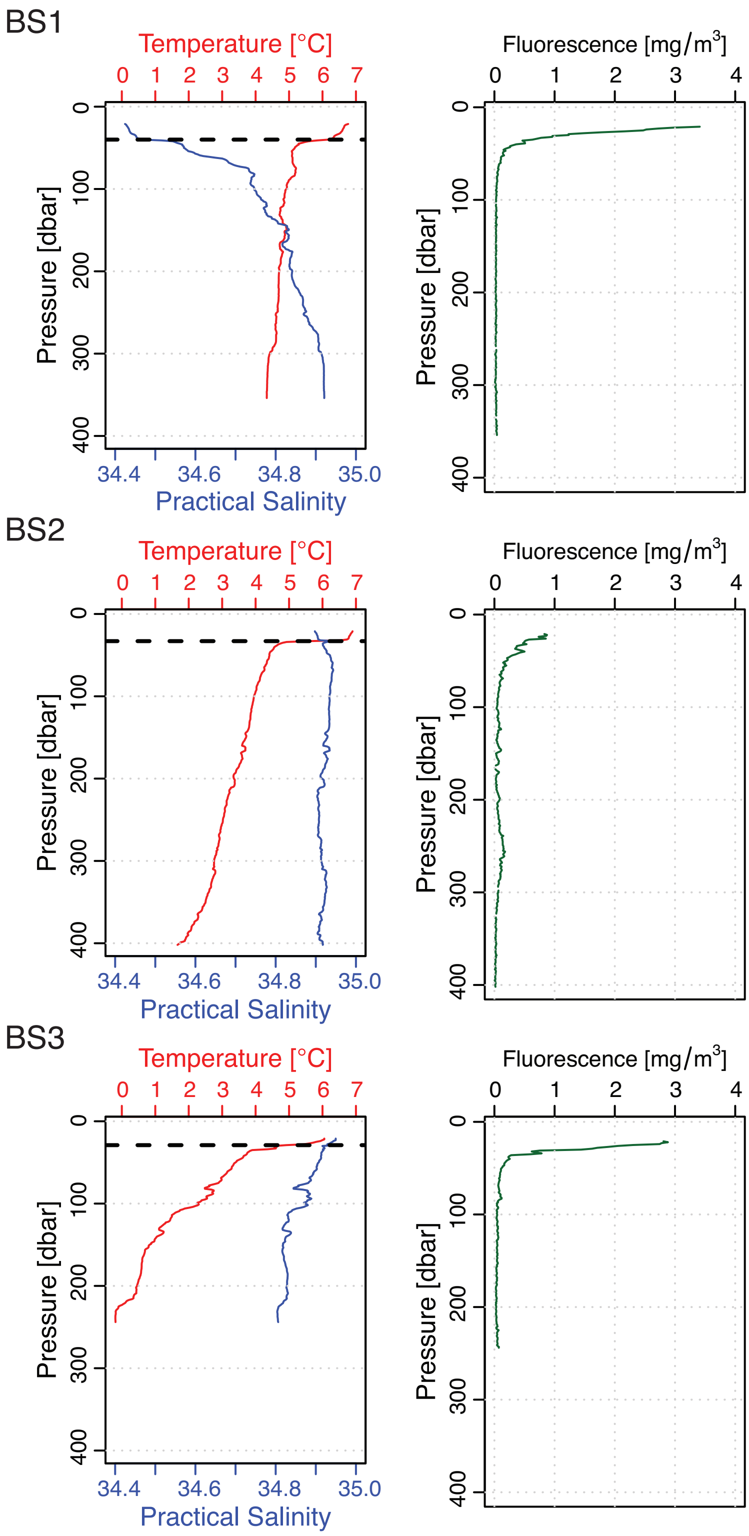

Supplementary Figure S3. Water column profiles of potential temperature, practical salinity and in situ fluorescence obtained from CTD casts at Barents Sea stations. Dashed line indicates density dependent mixed layer depth.

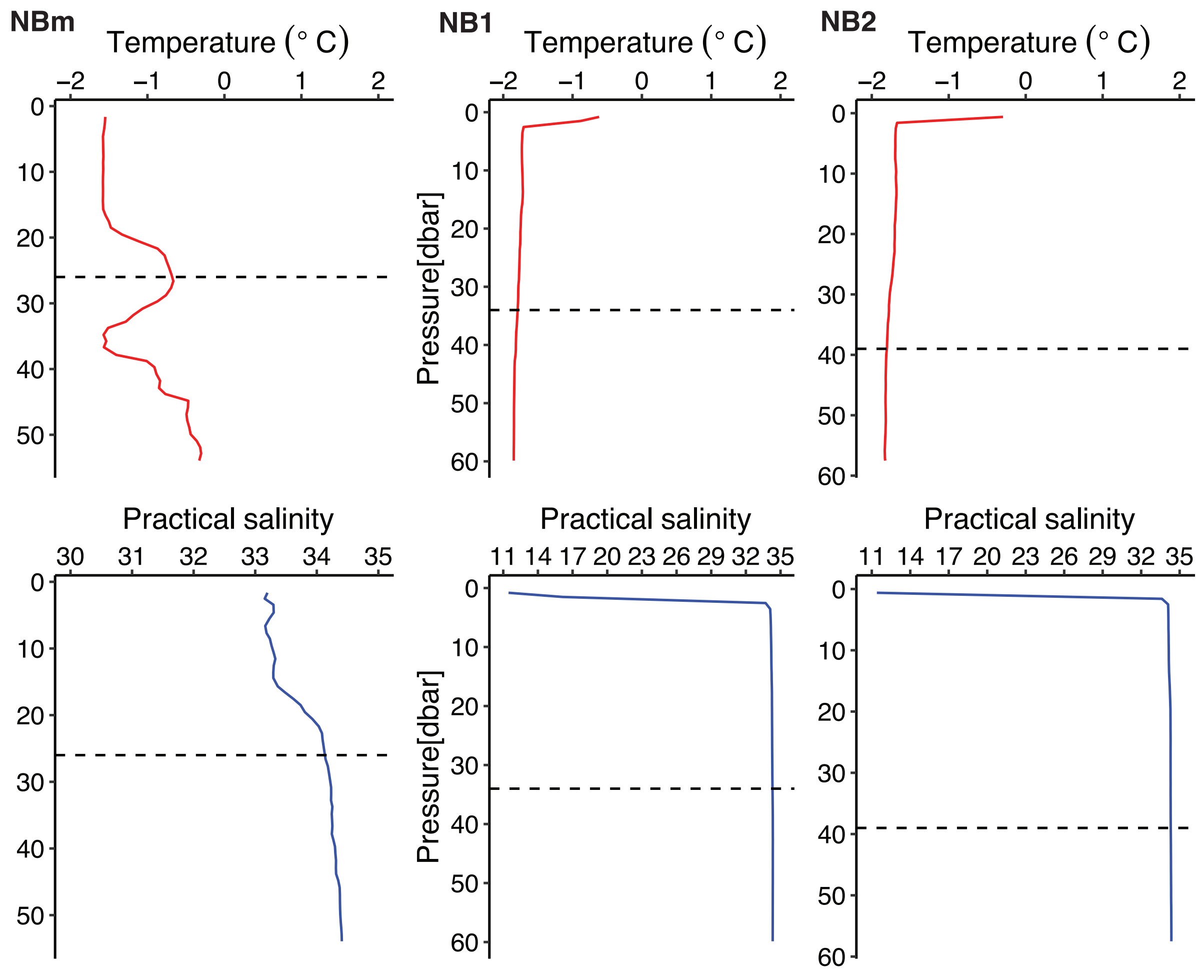

Supplementary Figure S4. Surface water profiles of potential temperature and practical salinity obtained from CTD casts at Nansen Basin margin (NBm) and Nansen Basin (NB1 and NB2) stations. Dashed line indicates density dependent mixed layer depth.

Supplementary Table S2. Results of Tukey's multiple comparison test between each sample on observed number of ASVs.

Station.contrast	null.value	estimate	std.error	statistic	adj.p.value	Data.set	Alpha.metric
BS2 - BS1	0	25,0	11,417	2,190	0,358	16S	Observed #ASVs
BS3 - BS1	0	10,3	11,417	0,905	0,986	16S	Observed #ASVs
NBm - BS1	0	48,2	11,417	4,219	0,001	16S	Observed #ASVs
NB1_0.5 - BS1	0	129,2	11,417	11,314	0,000	16S	Observed #ASVs
NB1_20 - BS1	0	163,0	11,417	14,277	0,000	16S	Observed #ASVs
NB2_0.5 - BS1	0	147,0	11,417	12,876	0,000	16S	Observed #ASVs
NB2_20 - BS1	0	189,3	11,417	16,584	0,000	16S	Observed #ASVs
BS3 - BS2	0	-14,7	11,417	-1,285	0,905	16S	Observed #ASVs
NBm - BS2	0	23,2	11,417	2,029	0,462	16S	Observed #ASVs
NB1_0.5 - BS2	0	104,2	11,417	9,124	0,000	16S	Observed #ASVs
NB1_20 - BS2	0	138,0	11,417	12,088	0,000	16S	Observed #ASVs
NB2_0.5 - BS2	0	122,0	11,417	10,686	0,000	16S	Observed #ASVs
NB2_20 - BS2	0	164,3	11,417	14,394	0,000	16S	Observed #ASVs
NBm - BS3	0	37,8	11,417	3,314	0,021	16S	Observed #ASVs
NB1_0.5 - BS3	0	118,8	11,417	10,409	0,000	16S	Observed #ASVs
NB1_20 - BS3	0	152,7	11,417	13,372	0,000	16S	Observed #ASVs
NB2_0.5 - BS3	0	136,7	11,417	11,971	0,000	16S	Observed #ASVs
NB2_20 - BS3	0	179,0	11,417	15,679	0,000	16S	Observed #ASVs
NB1_0.5 - NBm	0	81,0	11,417	7,095	0,000	16S	Observed #ASVs
NB1_20 - NBm	0	114,8	11,417	10,058	0,000	16S	Observed #ASVs
NB2_0.5 - NBm	0	98,8	11,417	8,657	0,000	16S	Observed #ASVs
NB2_20 - NBm	0	141,2	11,417	12,365	0,000	16S	Observed #ASVs
NB1_20 - NB1_0.5	0	33,8	11,417	2,963	0,060	16S	Observed #ASVs
NB2_0.5 - NB1_0.5	0	17,8	11,417	1,562	0,773	16S	Observed #ASVs
NB2_20 - NB1_0.5	0	60,2	11,417	5,270	0,000	16S	Observed #ASVs
NB2_0.5 - NB1_20	0	-16,0	11,417	-1,401	0,857	16S	Observed #ASVs
NB2_20 - NB1_20	0	26,3	11,417	2,307	0,290	16S	Observed #ASVs
NB2_20 - NB2_0.5	0	42,3	11,417	3,708	0,005	16S	Observed #ASVs
BS2 - BS1	0	81,8	17,177	4,764	0,000	18S	Observed #ASVs
BS3 - BS1	0	41,3	17,177	2,406	0,238	18S	Observed #ASVs
NBm - BS1	0	-21,3	17,177	-1,242	0,919	18S	Observed #ASVs
NB1_0.5 - BS1	0	42,3	17,177	2,464	0,211	18S	Observed #ASVs
NB1_20 - BS1	0	24,8	17,177	1,446	0,836	18S	Observed #ASVs
NB2_0.5 - BS1	0	49,3	17,177	2,872	0,078	18S	Observed #ASVs
NB2_20 - BS1	0	17,0	17,177	0,990	0,976	18S	Observed #ASVs
BS3 - BS2	0	-40,5	17,177	-2,358	0,263	18S	Observed #ASVs
NBm - BS2	0	-103,2	17,177	-6,006	0,000	18S	Observed #ASVs
NB1_0.5 - BS2	0	-39,5	17,177	-2,300	0,294	18S	Observed #ASVs
NB1_20 - BS2	0	-57,0	17,177	-3,318	0,021	18S	Observed #ASVs
NB2_0.5 - BS2	0	-32,5	17,177	-1,892	0,556	18S	Observed #ASVs
NB2_20 - BS2	0	-64,8	17,177	-3,774	0,004	18S	Observed #ASVs
NBm - BS3	0	-62,7	17,177	-3,648	0,006	18S	Observed #ASVs
NB1_0.5 - BS3	0	1,0	17,177	0,058	1,000	18S	Observed #ASVs
NB1_20 - BS3	0	-16,5	17,177	-0,961	0,980	18S	Observed #ASVs
NB2_0.5 - BS3	0	8,0	17,177	0,466	1,000	18S	Observed #ASVs

NB2_20 - BS3	0	-24,3	17,177	-1,417	0,850	18S	Observed #ASVs
NB1_0.5 - NBm	0	63,7	17,177	3,706	0,005	18S	Observed #ASVs
NB1_20 - NBm	0	46,2	17,177	2,688	0,126	18S	Observed #ASVs
NB2_0.5 - NBm	0	70,7	17,177	4,114	0,001	18S	Observed #ASVs
NB2_20 - NBm	0	38,3	17,177	2,232	0,332	18S	Observed #ASVs
NB1_20 - NB1_0.5	0	-17,5	17,177	-1,019	0,972	18S	Observed #ASVs
NB2_0.5 - NB1_0.5	0	7,0	17,177	0,408	1,000	18S	Observed #ASVs
NB2_20 - NB1_0.5	0	-25,3	17,177	-1,475	0,821	18S	Observed #ASVs
NB2_0.5 - NB1_20	0	24,5	17,177	1,426	0,845	18S	Observed #ASVs
NB2_20 - NB1_20	0	-7,8	17,177	-0,456	1,000	18S	Observed #ASVs
NB2_20 - NB2_0.5	0	-32,3	17,177	-1,882	0,563	18S	Observed #ASVs

Supplementary Table S3. Results of Tukey’s multiple comparison test between each sample on Faith’s Phylogenetic Diversity (PD).

Station.contrast	null.value	estimate	std.error	statistic	adj.p.value	Data.set	Alpha.metric
BS2 - BS1	0	6,9	1,214	5,703	0,000	16S	Faith's PD
BS3 - BS1	0	4,3	1,214	3,523	0,010	16S	Faith's PD
NBm - BS1	0	4,0	1,214	3,320	0,020	16S	Faith's PD
NB1_0.5 - BS1	0	17,9	1,214	14,762	0,000	16S	Faith's PD
NB1_20 - BS1	0	14,9	1,214	12,303	0,000	16S	Faith's PD
NB2_0.5 - BS1	0	15,0	1,214	12,349	0,000	16S	Faith's PD
NB2_20 - BS1	0	14,5	1,214	11,944	0,000	16S	Faith's PD
BS3 - BS2	0	-2,6	1,214	-2,180	0,363	16S	Faith's PD
NBm - BS2	0	-2,9	1,214	-2,382	0,250	16S	Faith's PD
NB1_0.5 - BS2	0	11,0	1,214	9,059	0,000	16S	Faith's PD
NB1_20 - BS2	0	8,0	1,214	6,600	0,000	16S	Faith's PD
NB2_0.5 - BS2	0	8,1	1,214	6,647	0,000	16S	Faith's PD
NB2_20 - BS2	0	7,6	1,214	6,242	0,000	16S	Faith's PD
NBm - BS3	0	-0,2	1,214	-0,202	1,000	16S	Faith's PD
NB1_0.5 - BS3	0	13,6	1,214	11,239	0,000	16S	Faith's PD
NB1_20 - BS3	0	10,7	1,214	8,780	0,000	16S	Faith's PD
NB2_0.5 - BS3	0	10,7	1,214	8,827	0,000	16S	Faith's PD
NB2_20 - BS3	0	10,2	1,214	8,422	0,000	16S	Faith's PD
NB1_0.5 - NBm	0	13,9	1,214	11,441	0,000	16S	Faith's PD
NB1_20 - NBm	0	10,9	1,214	8,983	0,000	16S	Faith's PD
NB2_0.5 - NBm	0	11,0	1,214	9,029	0,000	16S	Faith's PD
NB2_20 - NBm	0	10,5	1,214	8,624	0,000	16S	Faith's PD
NB1_20 - NB1_0.5	0	-3,0	1,214	-2,458	0,213	16S	Faith's PD
NB2_0.5 - NB1_0.5	0	-2,9	1,214	-2,412	0,235	16S	Faith's PD
NB2_20 - NB1_0.5	0	-3,4	1,214	-2,817	0,091	16S	Faith's PD
NB2_0.5 - NB1_20	0	0,1	1,214	0,046	1,000	16S	Faith's PD
NB2_20 - NB1_20	0	-0,4	1,214	-0,359	1,000	16S	Faith's PD
NB2_20 - NB2_0.5	0	-0,5	1,214	-0,405	1,000	16S	Faith's PD
BS2 - BS1	0	3,0	1,174	2,592	0,159	18S	Faith's PD
BS3 - BS1	0	2,9	1,174	2,460	0,213	18S	Faith's PD

NBm - BS1	0	-1,7	1,174	-1,445	0,836	18S	Faith's PD
NB1_0.5 - BS1	0	3,8	1,174	3,255	0,025	18S	Faith's PD
NB1_20 - BS1	0	1,1	1,174	0,970	0,979	18S	Faith's PD
NB2_0.5 - BS1	0	3,0	1,174	2,542	0,177	18S	Faith's PD
NB2_20 - BS1	0	1,4	1,174	1,159	0,943	18S	Faith's PD
BS3 - BS2	0	-0,2	1,174	-0,132	1,000	18S	Faith's PD
NBm - BS2	0	-4,7	1,174	-4,036	0,001	18S	Faith's PD
NB1_0.5 - BS2	0	0,8	1,174	0,664	0,998	18S	Faith's PD
NB1_20 - BS2	0	-1,9	1,174	-1,622	0,737	18S	Faith's PD
NB2_0.5 - BS2	0	-0,1	1,174	-0,050	1,000	18S	Faith's PD
NB2_20 - BS2	0	-1,7	1,174	-1,433	0,842	18S	Faith's PD
NBm - BS3	0	-4,6	1,174	-3,905	0,002	18S	Faith's PD
NB1_0.5 - BS3	0	0,9	1,174	0,795	0,993	18S	Faith's PD
NB1_20 - BS3	0	-1,7	1,174	-1,490	0,813	18S	Faith's PD
NB2_0.5 - BS3	0	0,1	1,174	0,082	1,000	18S	Faith's PD
NB2_20 - BS3	0	-1,5	1,174	-1,301	0,899	18S	Faith's PD
NB1_0.5 - NBm	0	5,5	1,174	4,700	0,000	18S	Faith's PD
NB1_20 - NBm	0	2,8	1,174	2,415	0,235	18S	Faith's PD
NB2_0.5 - NBm	0	4,7	1,174	3,987	0,002	18S	Faith's PD
NB2_20 - NBm	0	3,1	1,174	2,604	0,154	18S	Faith's PD
NB1_20 - NB1_0.5	0	-2,7	1,174	-2,285	0,302	18S	Faith's PD
NB2_0.5 - NB1_0.5	0	-0,8	1,174	-0,713	0,997	18S	Faith's PD
NB2_20 - NB1_0.5	0	-2,5	1,174	-2,096	0,417	18S	Faith's PD
NB2_0.5 - NB1_20	0	1,8	1,174	1,572	0,767	18S	Faith's PD
NB2_20 - NB1_20	0	0,2	1,174	0,189	1,000	18S	Faith's PD
NB2_20 - NB2_0.5	0	-1,6	1,174	-1,383	0,865	18S	Faith's PD

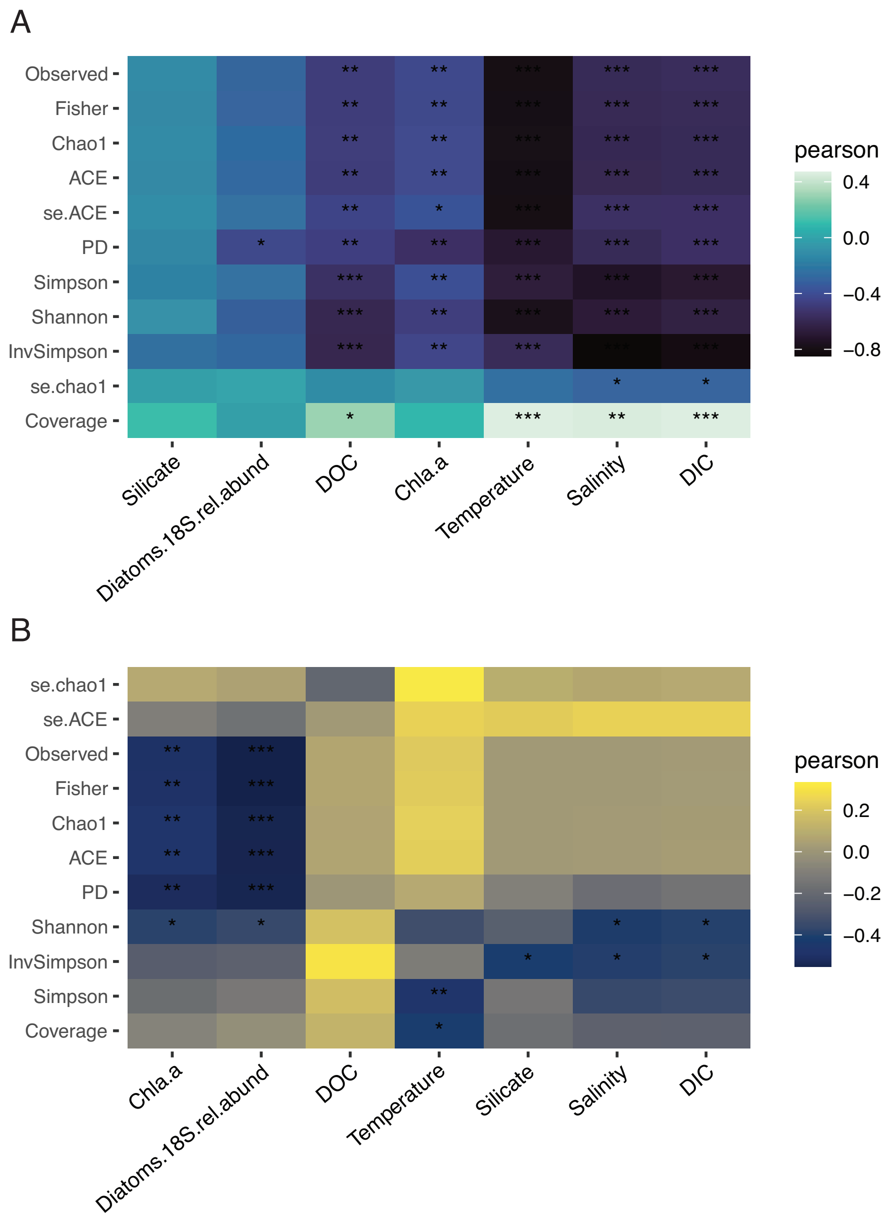

Supplementary Figure S5. Pearson’s correlation between prokaryotic (A) and microeukaryotic (B) alpha diversity metrics and environmental factors. Colors indicate Pearson’s correlation coefficient according to color legend and level of significance is marked with ★ (★, $p \leq 0.05$; ★★, $p \leq 0.01$; ★★★, $p \leq 0.001$).
